# Reference-Free Population Genomics from Next-Generation Transcriptome Data and the Vertebrate–Invertebrate Gap

**DOI:** 10.1371/journal.pgen.1003457

**Published:** 2013-04-11

**Authors:** Philippe Gayral, José Melo-Ferreira, Sylvain Glémin, Nicolas Bierne, Miguel Carneiro, Benoit Nabholz, Joao M. Lourenco, Paulo C. Alves, Marion Ballenghien, Nicolas Faivre, Khalid Belkhir, Vincent Cahais, Etienne Loire, Aurélien Bernard, Nicolas Galtier

**Affiliations:** 1 Université Montpellier 2, CNRS UMR 5554, Institut des Sciences de l'Evolution de Montpellier, Montpellier, France; 2 Université François Rabelais, CNRS UMR 7261, Institut de Recherche sur la Biologie de l'Insecte, Faculté des Sciences et Techniques, Tours, France; 3 Centro de Investigação em Biodiversidade e Recursos Genéticos (CIBIO), Universidade do Porto, InBIO Laboratório Associado, Campus Agrário de Vairão, Vairão, Portugal; 4 Departamento de Biologia, Faculdade de Ciências, Universidade do Porto, Porto, Portugal; 5 Wildlife Biology Program, College of Forestry and Conservation, University of Montana, Missoula, Montana, United States of America; University of Cambridge, United Kingdom

## Abstract

In animals, the population genomic literature is dominated by two taxa, namely mammals and drosophilids, in which fully sequenced, well-annotated genomes have been available for years. Data from other metazoan phyla are scarce, probably because the vast majority of living species still lack a closely related reference genome. Here we achieve *de novo*, reference-free population genomic analysis from wild samples in five non-model animal species, based on next-generation sequencing transcriptome data. We introduce a pipe-line for cDNA assembly, read mapping, SNP/genotype calling, and data cleaning, with specific focus on the issue of hidden paralogy detection. In two species for which a reference genome is available, similar results were obtained whether the reference was used or not, demonstrating the robustness of our *de novo* inferences. The population genomic profile of a hare, a turtle, an oyster, a tunicate, and a termite were found to be intermediate between those of human and Drosophila, indicating that the discordant genomic diversity patterns that have been reported between these two species do not reflect a generalized vertebrate versus invertebrate gap. The genomic average diversity was generally higher in invertebrates than in vertebrates (with the notable exception of termite), in agreement with the notion that population size tends to be larger in the former than in the latter. The non-synonymous to synonymous ratio, however, did not differ significantly between vertebrates and invertebrates, even though it was negatively correlated with genetic diversity within each of the two groups. This study opens promising perspective regarding genome-wide population analyses of non-model organisms and the influence of population size on non-synonymous versus synonymous diversity.

## Author Summary

The analysis of genomic variation between individuals of a given species has so far been restricted to a small number of model organisms, such as human and fruitfly, for which a fully sequenced, well-annotated reference genome was available. Here we show that, thanks to next-generation high-throughput sequencing technologies and appropriate genotype-calling methods, *de novo* population genomic analysis is possible in absence of a reference genome. We characterize the genomic level of neutral and selected polymorphism in five non-model animal species, two vertebrates and three invertebrates, paying particular attention to the treatment of multi-copy genes. The analyses demonstrate the influence of population size on genetic diversity in animals, the two vertebrates (hare, turtle) and the social insect (termite) being less polymorphic than the two marine invertebrates (oyster, tunicate) in our sample. Interestingly, genomic indicators of the efficiency of natural selection, both purifying and adaptive, did not vary in a simple, predictable way across organisms. These results prove the value of a diversified sampling of species when it comes to understand the determinants of genome evolutionary dynamics.

## Introduction

Population genomics, the analysis of within-species, genome-wide patterns of molecular variation, is a promising area of research, both applied and fundamental [1]. So far such studies have essentially been restricted to model organisms such as yeast [2] and Arabidopsis [3], in which a well-annotated, completely sequenced genome is available. In animals, the population genomic literature has long been dominated by drosophila and human (e.g. [4], [5]). Interestingly, these two species yielded very different patterns of genome variation. The per-site average synonymous nucleotide heterozygosity (π_S_), for instance, is roughly twenty times as high in *Drosophila melanogaster* (π_S_∼0.02 [6]) as in *Homo sapiens* (π_S_∼0.001 [7]) coding sequences. The ratio of non-synonymous to synonymous polymorphisms (π_N_/π_S_) is substantially lower, and the estimated proportion of adaptive amino-acid evolution (α) substantially higher, in *D. melanogaster* than in *H. sapiens* [8]–[12]. These distinctive patterns are interpreted as reflecting differences in effective population size (*N*_e_) between human, a large vertebrate, and drosophila, a tiny invertebrate. A small *N*_e_ in human would explain the relatively low level of genetic diversity in this species, as well as a reduced efficacy of natural selection due to enhanced genetic drift, which would increase the probability of segregation of slightly deleterious mutations (hence the higher π_N_/π_S_), and decrease the probability of fixation of adaptive ones (hence the lower α [13], [14]).

The human-drosophila contrast, however instructive it has been for molecular evolutionary research, is a comparison between just two species, out of the millions of existing animals. It is unclear whether the same picture would have been reached if a distinct vertebrate and a distinct invertebrate species had been sampled. Population genomic statistics in *D. simulans* were found to be essentially similar to those of *D. melanogaster* [15], and the central chimpanzee (*Pan troglodytes*), although genetically more diverse than *H. sapiens*, showed genomic patterns consistent with a relatively low-*N*_e_ species [16]. These are knowledgeable corroborations, but from species very closely related to *D. melanogaster* or *H. sapiens*. A very high amount of synonymous diversity and a very low π_N_/π_S_ ratio were reported in the tunicate *Ciona intestinalis* B [17]. This was interpreted as reflecting both a high mutation rate and large population size in this marine invertebrate species. Based on a small number of markers but many species, it was found that the average nuclear genetic diversity is higher in invertebrates than in vertebrates, and in marine than in terrestrial species [18], even though the difference is lower than expected from the neutral theory [19]. The influence of *N*_e_ was also invoked to explain the variations in non-synonymous to synonymous substitution rate between species of mammals [20], [21], and between populations of mice [22] and sunflower [23].

A recent population genomic study of the European rabbit (*Oryctolagus cuniculus*), however, revealed large amounts of genetic diversity, and a π_N_/π_S_ ratio similar to those measured in Drosophila [24]. Although perhaps abundant, rabbits, being vertebrates, are among the 5% largest living animal species. Observing a very low π_N_/π_S_ ratio in this species is somehow surprising according to the population size hypothesis, knowing that density and body mass tend to be negatively correlated across species (e.g. [25]). Still in mammals, relatively high levels of genomic polymorphism in endangered primate species were recently reported [26], again questioning the link between current abundance and population genomic patterns. It should be noted that what matters regarding molecular evolution is the long-term *N*_e_, averaged over thousands to millions of generations. It is therefore perhaps not so surprising that the *N*_e_ effect in mammals is not correctly predicted by species conservation status, as discussed in reference [26]. At any rate, the sample of metazoan species for which population genomic data are available is still quite small, and highly biased towards mammals. Genome-wide studies of additional species from various phyla appear needed to confirm or infirm the role of *N*_e_ in animal molecular evolution, and to explore variations of within-species genomic diversity across the phylogenetic and ecological dimensions.

Next-Generation Sequencing (NGS) technologies potentially offer the opportunity to gather population genomic data in non-model organisms, in the absence of prior knowledge, at affordable cost. Genomes in animals can be large, highly repetitive and, consequently, difficult to assemble. The transcriptome appears as a valuable alternative target [26]. Transcriptomics gives access to large numbers of genes at relatively low cost, plus information about gene expression levels [27]–[29], with potential applications for SNP discovery and speciation genomics [30]–[32]. However, unlike PCR-based techniques, NGS does not return alleles or genotypes at well-defined loci, but rather large amounts of mixed, noisy, anonymous sequence reads. Extracting proper population genetic information from such data is a challenge, both conceptually and computationally. Starting from raw NGS transcriptomic data, one must assemble predicted cDNA, map reads, call single nucleotide polymorphisms (SNPs) and genotypes, and calculate population genetics statistics. Each of these steps requires appropriate methods and data-cleaning strategies to cope with paralogous gene copies, unequal expression level across genes, alternative splicing, transcription errors, sequencing errors and missing data, among other problems. Obviously, the whole task is especially difficult in the absence of a well-assembled reference genome.

Here we introduce a pipeline for *de novo* transcriptome-based NGS population genomics, which is applied to newly-generated data from five animal species – two vertebrates and three invertebrates. Based on samples of eight to ten individuals caught in the wild, we identify between ∼4,500 and ∼17,000 SNPs per species, from ∼2000–3500 distinct nuclear protein-coding genes. For each species, we separate synonymous versus non-synonymous variants, and estimate the level of genetic polymorphism, the amount of divergence to a closely-related outgroup, site-frequency spectra, and adaptive evolutionary rates. We assess the robustness of these statistics to various SNP-calling and data cleaning options, and to the presence/absence of a reference genome, paying specific attention to the removal of spurious SNPs due to hidden paralogy. Then we focus on the between-species variation in the average synonymous and non-synonymous levels of within-species diversity. Our expectation is that small-*N*_e_ species should show a lower π_N_, a lower π_S_, and a higher π_N_/π_S_ ratio than large-*N*_e_ species. This is because genetic drift, which is enhanced in small populations, is expected to reduce the neutral and selected levels of genomic diversity, but to increase the relative probability of slightly deleterious, non-synonymous mutations (relatively to neutral, synonymous mutations) segregating at observable frequency. Our analyses suggest that the vertebrate versus invertebrate contrast is not an obvious predictor of *N*_e_ from a molecular evolutionary viewpoint.

## Results

### Target species

Table 1 lists the five species studied in this work. The urochordate *Ciona intestinalis* is a model organism for evo-devo research [33]. The existence of two cryptic species, called A and B, has recently been discovered [34], [35]. *C. intestinalis* A, which occupies the Pacific Ocean and the Mediterranean Sea, was taken as the focal species in this study. The flat oyster *Ostrea edulis* is a marine bivalve of economic interest, which lives in the Eastern Atlantic coasts. *C. intestinalis* and *O. edulis* belong to two phyla, tunicates and bivalves, in which very high levels of within-species genetic diversity have been reported [17]–[19], [36]–[38]. The Iberian hare *Lepus granatensis* has attracted the attention as a model taxon for phylogeographic analysis and the study of speciation and reticulate evolution [39]. Its geographic range is limited to Iberia. The European pond turtle *Emys orbicularis* occurs in freshwater environments in Europe [40]. Both *L. granatensis* and *E. orbicularis* are terrestrial, medium-sized vertebrates, for which a relatively low *N*_e_ can be expected. The subterranean termite *Reticulitermes grassei*, finally, is a eusocial termite species occurring in Spain and south-west France, feeding on wood, and causing damage to human habitations. *R. grassei* is a small invertebrate, by far the smallest of the five species analyzed here. However, its effective population size is presumably highly reduced by eusociality – few individuals per colony contribute to reproduction. In the rest of the article, these five species will be designated as ciona, oyster, hare, turtle and termite, respectively.

**Table 1:** Illumina data sets used in this study.

Focal species	Outgroup	#Individuals (focal+outgroup)	Megareads (all individuals)	Megabases (per individual)
*Ciona intestinalis A* (ciona)	*C. intestinalis B*	10+10	139	677
*Ostrea edulis* (oyster)	*O. chilensis*	10+2	63	471
*Lepus granatensis* (hare)	*L. americanus*	10+1	66	544
*Emys orbicularis* (turtle)	*Trachemys scripta*	10+2	94	710
*Reticulitermes grassei* (termite)	*R. flavipes*	9+2	250	1069

A reference genome and transcriptome is available for two species of our panel, namely ciona, which was fully sequenced [41], and hare, which is closely related (∼5% divergence) to the fully-sequenced rabbit, *O. cuniculus* [24]. For these two species, reference-free population genomic inferences were compared to reference-based ones. For each of the five focal species, a closely-related outgroup was included in the study in order to perform divergence analyses. The outgroup was taken from the same genus as the focal species, except for the turtle, in which the outgroup was the pond slider *Trachemys scripta* (Table 1).

### cDNA assembly, read mapping, and genotype-calling

Table 1 describes the NGS data sets generated in this analysis. Nine to ten individuals per focal species and two to eight individuals per outgroup species were analysed. An average 7.85 millions single-ended illumina reads of mean length 89 were obtained per individual. In oyster, termite, hare, and turtle, 454 analysis of one or a pool of individuals provided an additional ∼500,000 reads of average length 306. Roughly 50% of the data were newly generated for this study. The other 50%, i.e., eight individuals each of ciona (B species), oyster, hare and turtle, were previously used to investigate various cDNA assembling strategies [42].

**Figure 1:**
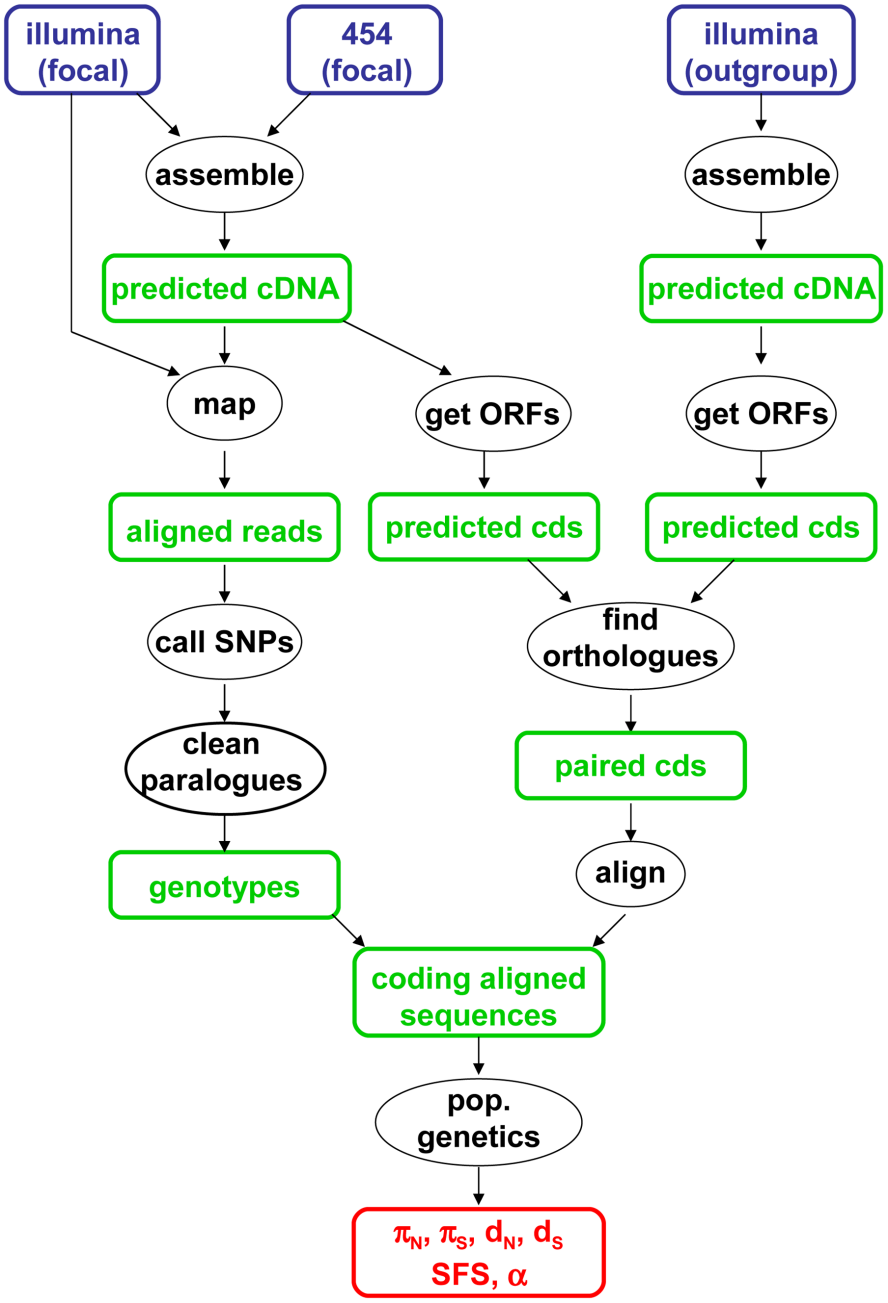
Main data analysis pipeline used in this study.

The data analysis pipeline is illustrated by Figure 1, and fully described in the Material & Methods section. Depending on the species, between 28,000 and 85,000 contigs were generated by a combination of Abyss and Cap3. Illumina reads were mapped onto the predicted cDNAs using BWA. Genotypes were called using program reads2snps, which implements the maximum likelihood framework introduced by Tsagkogeorga et al. [17], in which the per-contig error rate is estimated assuming a multinomial distribution of read counts and the Hardy-Weinberg equilibrium. When the posterior probability of the best-supported genotype (either homozygote or heterozygote) was below 0.95, the position was coded as missing data. Classical population genomic statistics were calculated based on these predicted genotypes, after various data cleaning steps, using custom-witten C++ programs. The number of contigs available for population genomic analyses – i.e., contigs which passed the coverage and ORF length filters – varied among species from 1978 to 3661. Note that the 454 reads were only used at the assembly step, not for individual genotyping.

### Paralogue filtering

In the genotype-calling procedure described above, we assume that all the reads that map to a given position correspond to a single locus. It might be, however, that reads from distinct loci map to the same place. This is expected to occur in cases of undetected paralogy, copy number variation, and repetitive genomes. In such cases, variation between paralogues might result in spurious heterozygous genotype calls. We introduced a new test to detect and clean these spurious heterozygotes. Briefly, the rationale is to compare the likelihood of a model assuming one bi-allelic locus with the likelihood of a model assuming two bi-allelic loci, both carrying the same two alleles (see Material and methods and Text S1 for details). Among the sites at which at least one heterozygous genotype was called, those for which the paralogy test was significant (p-val<0.001) were discarded. Depending on the species, between 7% (ciona) and 37% (hare) of SNPs were detected as potential paralogues.

### Quality control analyses

Our major analyses involve comparison of population genetic statistics between species, and so it is important to be sure that these differences are due to real biological differences and not methodological artefacts. We first analysed the variations and impact of sequencing coverage across samples and genes. The average coverage of the analysed contigs varied from 5X to 15X across individuals and species after removal of potential PCR duplicates (Figure S1), oyster being slightly less covered, on average, than the other four species. The observed heterozygosity (i.e., the proportion of predicted heterozygous sites) was calculated for all individuals. Its relative level of variation among individuals was minimal in hare (0.0013–0.0018), and maximal in turtle (0.0003–0.0017). Importantly, this value was not correlated with the average sequencing depth in any of the five species – individuals for which large amounts of data were obtained were not more (or less) heterozygous, on average, than other individuals (Figure S1). The correlation coefficient of sequencing coverage across genes was typically above 0.9 for individuals from the same species, and declined when individuals from distinct species were compared, consistent with reference [26]. No correlation was found across species between the between-individual variance in sequencing depth and the mean or between-individual variance in heterozygosity (result not shown).

Then, in all five species, the contig containing the *cox1* mitochondrial gene was identified by BLAST and individually analysed. *Cox1* is a highly-expressed, haploid locus for which homozygous genotypes should be recovered if nuclear-encoded paralogs (the so-called “numt”) have been correctly filtered, and contamination between samples avoided. In turtle, ciona, oyster and termite, *cox1* genealogies revealed monophyletic species, and amounts of within-species mitochondrial diversity below 1% (Figure S2). Examining the predicted SNPs, we found a single (in oyster) predicted heterozygous genotype out of the ∼40,000 genotyped positions. The average proportion of heterozygous genotypes across individuals and positions in these four species was 4.10^−5^, i.e., very low.

In hare, the *cox1* tree revealed two divergent groups of *L. granatensis* haplotypes, of which one was more closely related to the arctic hare *Lepus timidus*. This is consistent with the documented introgression of *L. timidus* mitochondrial DNA into northern iberian populations of *L. granatensis* [39], [43]. A closer examination of the *cox1* contig analysed here revealed that it was a complex chimera, *i.e.*, a concatenation of fragments from the *granatensis* and *timidus* haplotypes, which are ∼10% divergent from each other. Six positions in this alignment contained unexpected heterozygous genotypes. Five of them were located close to (<30 bp away from) the boundary between a *granatensis* and a *timidus* fragment. The heterozygous genotypes correspond to low-coverage positions/individuals, which occurred when most reads from a specific individual had mapped to a distinct contig – the hare assembly included several other highly-covered contigs homologous to *cox1*, of length 200–460 bp. When a minimal coverage of 30X per individual, instead of 10X per individual, was required to call a genotype (our “high-coverage control”, see below), all the unexpected heterozygotes disappeared. We note that such a situation – two divergent, highly-expressed alleles coexisting in the population, with each individual carrying a single copy – is presumably very uncommon. The results of our main analyses were qualitatively unchanged when the three introgressed individuals were removed from the hare data set. To summarize, our analysis of the *Cox1* gene were consistent with previous knowledge regarding mtDNA evolution in the five target species, and revealed a satisfying behaviour of our genotype-calling procedure, in its basic or high-coverage version.

Finally, we investigated the geographic patterns of genetic variation the five analysed species by plotting between-individual genetic versus geographic distance (Figure S3). A clear isolation-by-distance pattern was detected in ciona, in which the Mediterranean and Californian samples were differentiated, and in turtle, in which some population substructure associated with Pleistocene glacial refugia is detected. The relationship was much weaker in oyster, and absent in hare and termite. These patterns are essentially consistent with the phylogeographic literature in these five species [40], [44]–[47], which is typically based on fewer loci but many more individuals than the current study. The concordance between these two sources of data provides additional corroboration for our inferred SNPs and genotypes.

### Robustness of population genetic estimates to methodological options

For each species, population genomic statistics were calculated and averaged across loci (Table 2, row A). Their robustness to various data cleaning/SNP calling options was examined in two species, ciona and hare, for which a full genome and a reference transcriptome are available.

**Table 2:** Robustness of population genomic statistics to SNP calling options.

	#contigs	av. lg	#SNPs	π_S_ (%)	π_N_ (%)	π_N_/π_S_	F_IS_
**ciona**:							
A. Main	3081	225	15 826	1.54±0.04	0.17±0.01	0.11±0.01	−0.04
B. High coverage	902	219	3 578	1.60	0.12	0.07	−0.02
C. Reference	2030	237	10 314	1.47	0.14	0.10	−0.03
D. No paralog filter	3 056	225	16 989	1.58	0.18	0.11	−0.06
E. Samtools	2 030	348	14 515	1.17	0.14	0.12	−0.02
**hare**:							
A. Main	2 624	276	7 261	0.41±0.03	0.06±0.01	0.15±0.02	−0.04
B. High coverage	790	264	1 611	0.43	0.05	0.12	−0.05
C. Reference	1 266	282	3 063	0.39	0.04	0.10	−0.04
D. No paralog filter	2 980	273	11 591	0.48	0.10	0.20	−0.14
E. Samtools	1 260	513	7 297	0.37	0.10	0.27	−0.03
**oyster**:							
A. Main	2 538	219	6 835	0.57	0.10	0.18	−0.05
E. Samtools	2 752	207	6 147	0.38	0.09	0.24	−0.04
**termite**:							
A. Main	8 086	366	8 697	0.12	0.02	0.19	0.12
E. Samtools	6 432	437	5 524	0.08	0.02	0.20	0.13
**turtle**:							
A. Main	2 013	243	4 634	0.45	0.07	0.16	0.17
E. Samtools	2 147	225	4 365	0.37	0.13	0.34	0.15

Estimates of π_N_ and, especially, π_S_ were reasonably robust to the high-coverage control, even though fewer SNPs were called with the increased coverage/quality requirement (Table 2, row B). This is because requiring a higher quality decreases not only the number of predicted SNPs, but also the number of predicted homozygous positions. The slightly lower π_N_/π_S_ ratio obtained from the high-coverage control might reflect a biological effect, i.e., stronger selective constraint on highly-expressed genes [48]. High levels of robustness were also obtained with respect to our “high-quality”, “threshold-free” and “clip-ends” controls (Table S2, row F, G, H).

Importantly, results were only weakly affected when reads were mapped on existing genomic references, rather than on predicted contigs (Table 2, row C). In ciona, both π_N_ and π_S_ were reduced by <10% in the reference-based control. In hare, the situation was a bit worse, with π_N_ being reduced by ∼30% when reads were mapped to the rabbit transcriptome, while π_S_ was unchanged. Note that in the case of hare, the reference is ∼5% divergent from our focal species, which might bias the sample towards evolutionarily conserved genes in the reference-based control. Taken together, the reference-based controls suggest that the uncertainty in cDNA prediction [42] does not impede *de novo* population genomic analysis from NGS transcriptomic data.

When potentially spurious SNPs due to undetected paralogy were not filtered out, the total number of analysed SNPs increased, as could have been expected (Table 2, row D). This change did not dramatically affect π_S_ and π_N_, but a lower (i.e., more negative) F_IS_ was obtained when the paralog filter was off. Negative F_IS_ denotes an excess of heterozygotes, as compared to the Hardy-Weinberg expectation. This is unexpected from natural population samples, in which population structure and inbreeding typically result in a deficiency, rather than an excess, of heterozygotes. The observed decrease in F_IS_ when the paralog filter was switched off suggests that erroneous SNPs/genotypes due to mapping problems are common, and that filtering them out is necessary. The slightly negative F_IS_ measured in our main ciona and hare analysis suggest that the filter does not entirely solve the problem.

**Figure 2:**
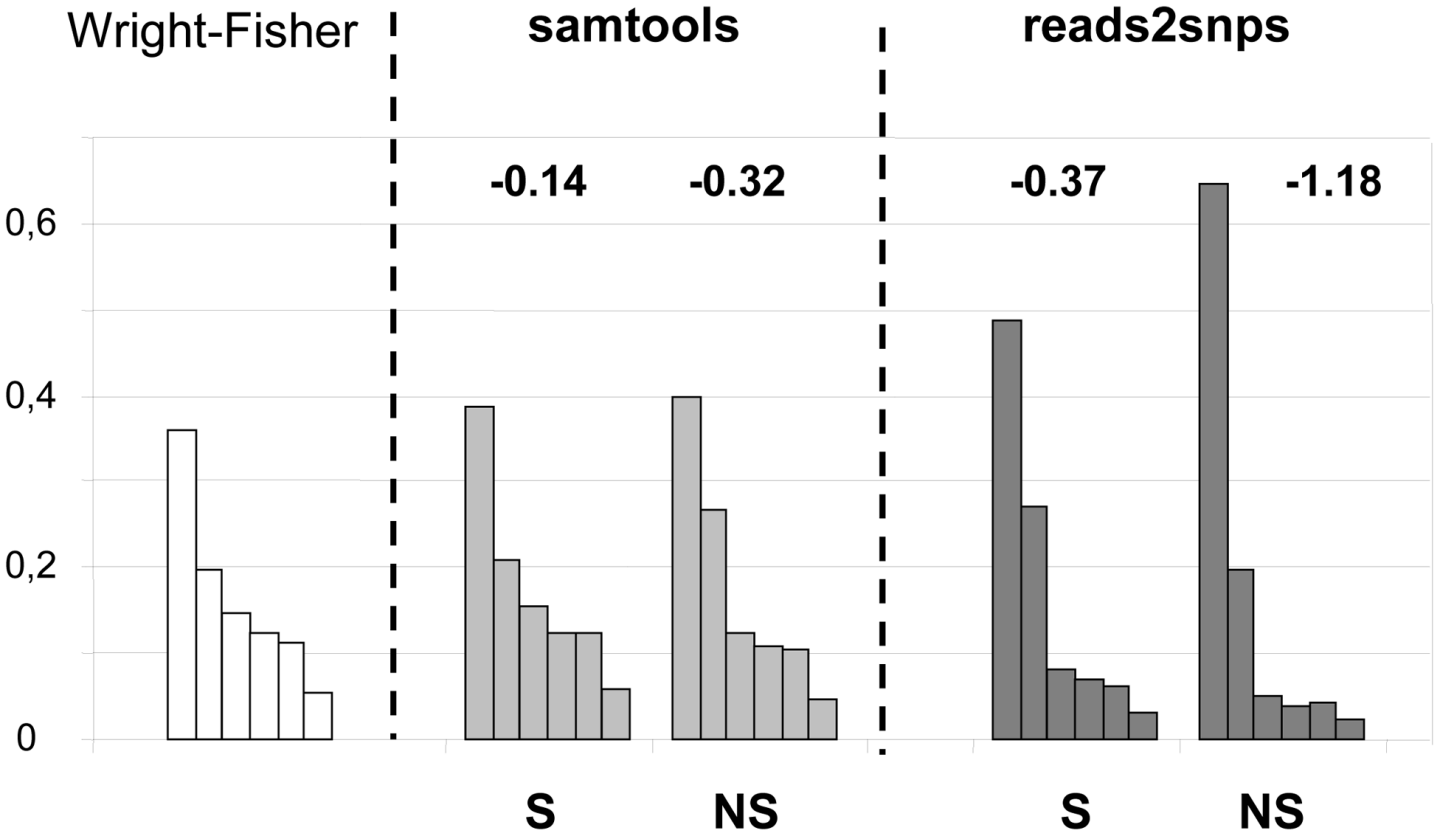
Synonymous and non-synonymous site-frequency spectra in the hare *Lepus granatensis*. Each histogram displays the distribution of minor allele frequency across SNPs (folded site-frequency spectrum) for a sampling size of 12 chromosomes. The left-most histogram is the expected spectrum for neutral sites in a Wright-Fisher population. The other four histograms were drawn from the data, calling SNPs with either Samtools or reads2snps, and separating non-synonymous (NS) from synonymous (S) positions. The number above each histogram is Tajima's D. This index is equal to zero in the Wright-Fisher case.

Our results were compared to an entirely different data analysis pipeline based on samtools [49] (Table 2, row E). The two approaches yielded similar results in ciona, but in hare π_S_ was slightly decreased, and π_N_/π_S_ substantially increased, when samtools was used. The same trend was observed in oyster, termite and turtle, to various extents (Table 2). To investigate further the causes of this discrepancy, we computed site frequency spectra (SFS) from the genotypes predicted by samtools versus reads2snps (our main analysis). Figure 2 displays the folded synonymous and non-synonymous SFS in hare. As far as reads2snps predictions were concerned, the proportion of low-frequency variants was higher in non-synonymous SNPs than in synonymous SNPs, as previously reported in human [13] and drosophila [50]. This is expected under the hypothesis of a prevalent influence of purifying selection on non-synonymous mutations. Such a pattern was not observed with the samtools-predicted SNPs, in which the synonymous and non-synonymous SFS were similar to each other, and similar to the SFS expected in a neutrally evolving, panmictic, Wright-Fisher population (Figure 2, left), in which the probability of observing a SNP at a derived allele frequency of *k* is proportional to 1/*k* [51]. The inferred SFS for the other four species are displayed in Figure S4. A pattern similar to the hare was observed in turtle and termite. In ciona and oyster, the contrast between the synonymous and non-synonymous spectra was weaker.

The samtools and reads2snps genotype callers differ in two main aspects. First, reads2snps does not make use of sequence quality data, and, instead, estimates the error rate, assumed to be constant across positions in a contig, from the data. When the analysis was restricted to high-quality reads only, reads2snps-based SFS were essentially unchanged (results not shown), which does not suggest that the treatment of sequencing errors is an issue here. Secondly, reads2snps places no explicit prior on the SFS, whereas the samtools caller uses a Wright-Fisher prior (equation 20 in [52]). This could explain the difference between reads2snps-predicted and samtools-predicted SFS, and especially the higher similarity of samtools-predicted SFS, both synonymous and non-synonymous, to the Wright-Fisher expectation, as reflected in Tajima's D values that are closer to zero (Figure 2, Figure S4).

Sequences from outgroup species were added to within-species alignments. Contigs showing extreme levels of synonymous divergence between focal and outgroup species (i.e., genes that exceeded the median *d*_S_ by two standard deviations or more) were considered as dubious and discarded. Outgroup inclusion resulted in a strong decrease in number of analysed contigs,and a slight reduction in estimated π_N_/π_S_ ratio (Table S2, row I). This presumably reflects a more accurate prediction of ORFs when data from two distinct species are available, and/or an increased level of selective constraint on the subset of genes for which orthology search was successful.

### Sampling bias and variance

**Figure 3:**
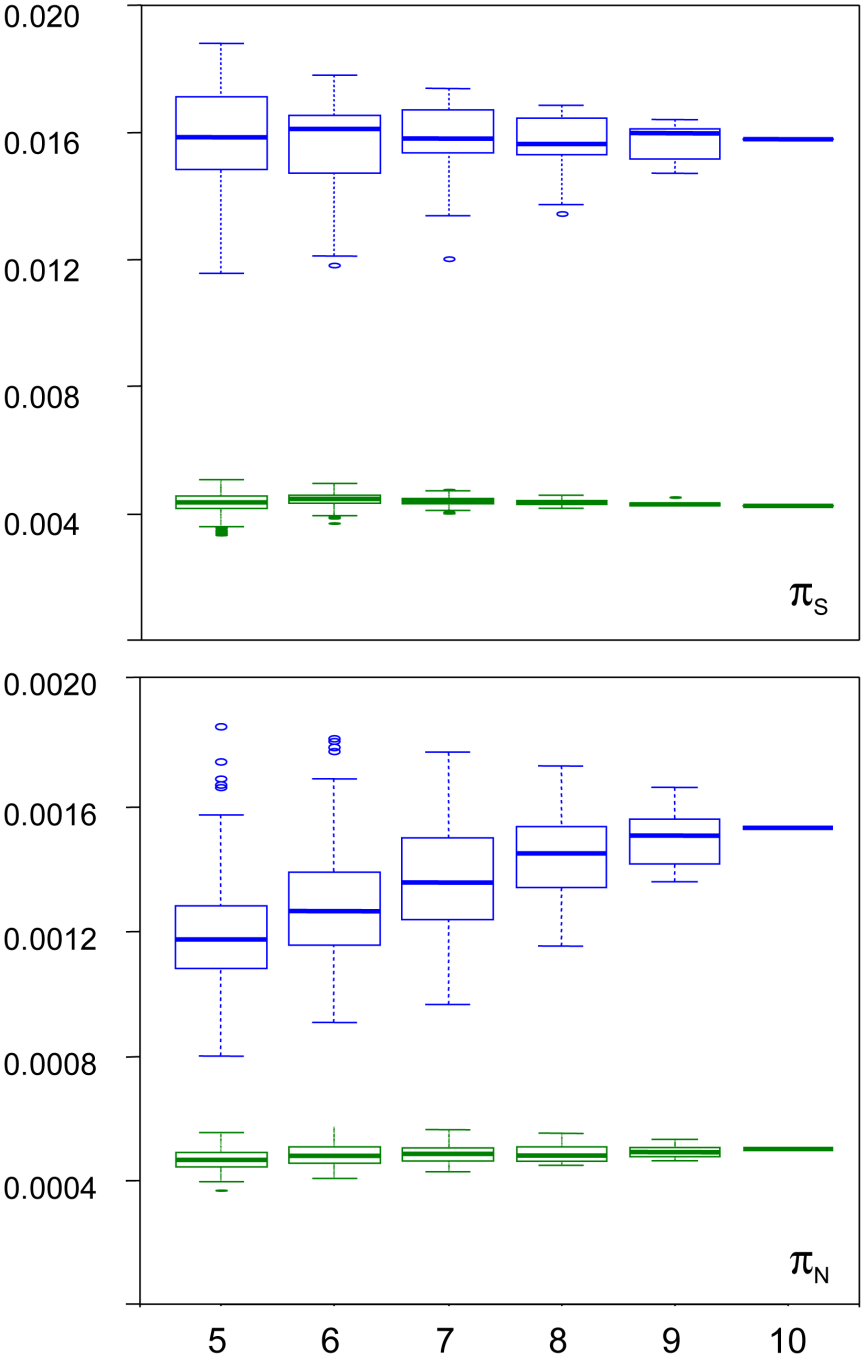
Sampling variance of π_N_ and π_S_ in the turtle *Emys orbicularis* and the tunicate *Ciona intestinalis* A. X-axis: size of individual sub-samples; Y-axis: box-plot of estimated synonymous (top) and non-synonymous (bottom) diversity in turtle (green) and ciona (blue).

We examined the robustness of our results to individual sampling. We generated random sub-samples of five to nine individuals (all combinations), and re-called SNPs and genotypes. Figure 3 shows the distribution of π_S_ and π_N_ across sub-samples, as a function of sub-sample size, in turtle (green) and ciona (blue). In turtle, no sampling bias was detected: the average estimated π_S_ and π_N_ did not vary with sub-sample size. The standard deviation across all sub-samples was 5% of the π_S_ estimate, and 7% of the π_N_ estimate. In ciona, no bias was detected for π_S_, but the estimated π_N_ slightly declined as sub-sample size decreased. The median π_N_ across sub-samples of five individuals was 23% lower than the estimate obtained from all ten individuals. The coefficient of variation was still relatively low for both π_S_ (8%) and π_N_ (12%). The hare pattern was similar to turtle, and the oyster and termite patterns similar to ciona. The reasons for a decline of π_N_ with sub-sample size in three species are unclear. The occurrence of this pattern does not appear related to the existence of population substructure (Figure S3). At any rate, this analysis indicates that our estimates of within-species synonymous and non-synonymous diversity are reasonably robust to sampling size, and that the sampling variance is well below the reported between-species differences.

### Synonymous versus non-synonymous polymorphism and divergence

Table 3 summarizes the population genomic statistics, calculated using our main settings, in the five species analysed in this study, with outgroup. The two vertebrates, hare and turtle, were less polymorphic than the three invertebrates, as could have been expected from intuition about population sizes. Ciona was the most polymorphic species of our panel. This is in line with the analysis of Tsagkogeorga et al, who reported an extremely high π_S_ in the congeneric *C. intestinalis* B [17]. Oyster, perhaps surprisingly, was not much more polymorphic than the two vertebrates as far as synonymous sites were concerned. A similar π_S_ estimate (0.07) was obtained by E. Harrang (personal communication) based on 37 loci Sanger-sequenced in a sample of 20 flat oysters. Termite, finally, was the least polymorphic species of the panel, consistent with the expectation of a reduced population size associated to eusociality.

**Table 3:** Coding sequence polymorphism and divergence patterns in five non-model animals.

species	#contigs	#SNPs	π_S_ (%)	π_N_ (%)	π_N_/π_S_	d_N_/d_S_	α	α_0.2_	α_EWK_	ω_A_
turtle	1 041	2 532	0.43±0.03	0.05±0.007	0.12±0.02	0.17±0.03	0.01±0.18	0.43±0.15	0.92	0.17
hare	524	2 054	0.38±0.04	0.05±0.008	0.12±0.02	0.15±0.03	−0.11±0.22	0.30±0.23	<0	<0
ciona	2 004	11 727	1.58±0.06	0.15±0.011	0.10±0.01	0.10±0.01	−0.28±0.10	0.10±0.11	0.34	0.04
termite	4 761	5 478	0.12±0.01	0.02±0.002	0.18±0.02	0.26±0.02	0.08±0.10	0.28±0.11	0.74	0.20
oyster	994	3 015	0.59±0.05	0.09±0.011	0.15±0.02	0.21±0.02	0.13±0.12	0.22±0.13	0.79	0.21

**Figure 4:**
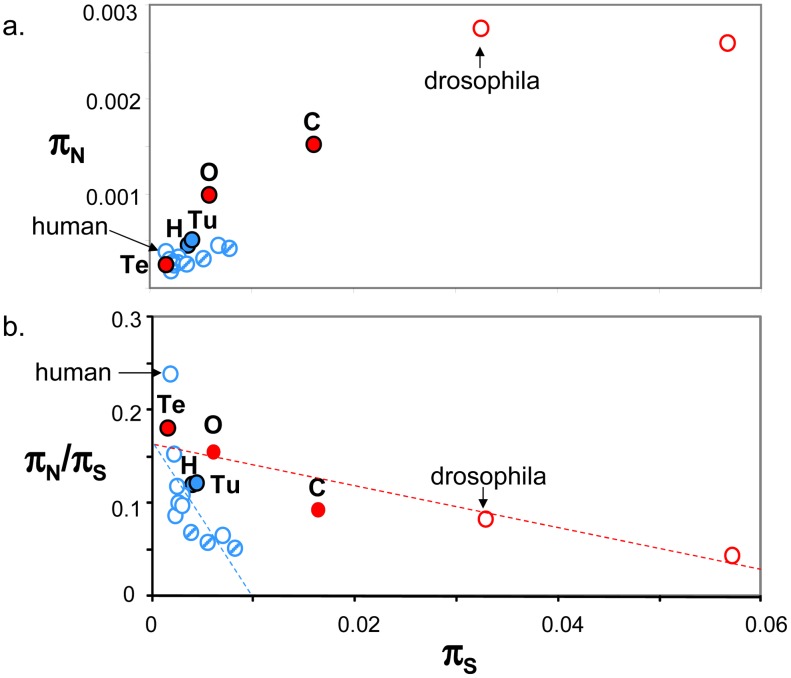
Published estimates of genome-wide π_S_, π_N_ and π_N_/π_S_ in animals. a. π_N_ as function of π_S_; b. π_N_/π_S_ as function of π_S_; Blue: vertebrates; Red: invertebrates; Full circles: species analysed in this study, designated by their upper-case initial (H: hare; Tu: turtle; O: oyster; Te: termite; C: ciona); Dashed blue circles: non-primate mammals (from left to right: mouse, tupaia, rabbit). Estimates were taken from Bustamante et al. 2005 (human), Hvilsom et al 2012 (chimpanzee), Carneiro et al 2012 (rabbit), Perry et al 2012 (other mammals), Begun et al 2007 (*D. simulans*) and Tsagkogeorga et al 2012 (*C. intestinalis* B = right-most circle).

Figure 4a plots genomic average π_N_ against genomic average π_S_ across 19 animal species for which such estimates are available from the literature ([10], [15]–[17], [24], [26], estimates obtained from at least four individuals caught in the wild and 1000 genes). This figure shows that the five species sampled here (closed circles) are intermediate between human and drosophila in terms of within-species diversity. Vertebrates (in blue), here represented by thirteen mammals (among which nine primates) and one turtle, showed an average π_S_ below 0.01, and an average π_N_ below 0.0006. More variance was detected within the group of invertebrate species, in which termite was a clear outlier. Both π_S_ and π_N_ reached in invertebrates values well above the maximal records of mammals and turtle. So a vertebrate versus invertebrate gap in genomic diversity is still apparent in Figure 4a, even though the contrast is not as sharp as suggested by the sole human versus drosophila comparison – and please note that the vertebrate taxon sampling is still highly biased towards mammals.

In Figure 4b, the π_N_/π_S_ ratio was plotted as a function of π_S_. A significant negative relationship was recovered both in vertebrates (r^2^ = 0.43, p-val<10^−5^, n = 14) and invertebrates (r^2^ = 0.86, p-val = 0.002, n = 5), in agreement with the hypothesis of a population size effect on the efficiency of purifying selection. However, the average π_N_/π_S_ ratio was not significantly higher in invertebrates than in vertebrates, and the correlation coefficient computed across all 19 species (r^2^ = 0.18) was not significantly different from zero. This is an intriguing result, which does not seem to accommodate well the idea of a *N_e_*-dependent π_N_/π_S_ ratio. Figure 4b was unchanged when the average π_S_ was calculated from one half of the contigs, and the average π_N_/π_S_ from the other half, thus removing any intrinsic dependence between the two variables (not shown). The ratio of non-synonymous to synonymous divergence, d_N_/d_S_, was also negatively correlated to π_S_, again in agreement with the hypothesis of a more efficient purifying selection in large populations (Figure S5).

The proportion of adaptive amino-acid substitutions, α, was estimated using two distinct methods based on the McDonald-Kreitman principle [8], and the (per synonymous substitution) rate of adaptive non-synonymous substitution, ω_a_, was computed too. Estimates of α varied from 0 to 0.9 among species and methods. In hare, the DoFE program returned a highly negative, aberrant value for α when the method of reference [53] was used. These estimates showed no obvious correlation with variations in effective population size. Neither α nor ω_a_ were found to be higher in invertebrates than in vertebrates when low-frequency variants were appropriately handled (Figure S5). Our data, therefore, do not bring support to the hypothesis of a higher adaptive rate in large-*N_e_* species, in contrast with several recent reports [22], [23], [54], [55]. We note that theoretical predictions are equivocal regarding the α/ω_a_/*N_e_* relationships: the adaptive rate itself appears to be strongly limited by linkage and hardly influenced by *N_e_* (assuming large enough populations and a constant supply of advantageous mutations [56], [57], and under purifying selection alone the α/*N_e_* relationship can be complex [58].

## Discussion

Here we show that population genomics is possible in absence of a reference genome, thanks to an appropriate treatment of NGS data. Based on *de novo* assembled contigs, predicted ORF, empirical estimation of sequencing/mapping error rate and statistical filtering of potential paralogs, we recovered estimates of the major population genomic statistics that were reasonably similar to the ones obtained using published genomic annotations. Our estimates were robust to various methodological options, including constraints on sequence quality and coverage, threshold-based versus threshold-free genotype calling, and sub-sampling of contigs or individuals. Our results are consistent with a larger amount of within-species genetic diversity in invertebrates than in vertebrates (with exceptions), but question the relevance of *N*_e_ as a determinant of the π_N_/π_S_ ratio and the adaptive substitution rate, which did not differ between vertebrates and invertebrates in our analysis.

### Methodological issues

From the several control steps we implemented, the most problematic issue we faced in this analysis was due to hidden paralogy, which manifested itself through spurious polymorphic positions at which many individuals, if not all, were heterozygous, and shared a common highly-expressed (and a common lowly expressed) allelic state. Dou et al. [59] recently highlighted this problem, and proposed a method to overcome it, based on the idea that sequencing coverage is expected to be higher in repeated than in unique genomic regions. This approach does not apply to transcriptomic data, in which coverage primarily reflects the level of gene expression, which is not only determined by gene copy number. We introduce a novel filtering method based on explicit modelling of the single versus multiple copy cases. Our analyses indicate that this method removes a large fraction of hidden paralogy instances, as suggested by the substantial reduction in heterozygote excess in ciona and hare. We presume that hidden paralogy will be identified as the major caveat of *de novo* population genomics in future research, as suggested by the relatively large amount of dubious SNPs that were filtered out in this analysis. Besides the paralogy issue, our results were quite robust to the several methodological options we tried. In particular, both π_S_ and individual heterozygosity were unrelated to sequencing depth (Table 2, high-coverage control and Figure S1) – a desirable property of NGS-based population genomic studies.

The two SNP-calling approaches we used yielded correlated (across species) but distinct results, with samtools predicting a lower SNP density than our reads2snps method. The two approaches differ in several aspects, including quality-based versus sequence-based estimation of the error rate, and whether a Wright-Fisher prior was used. Obviously, even slight differences in methodological design can have detectable consequences on the predicted genotypes, as suggested by the comparison between samtools-predicted and reads2snps-predicted site frequency spectra (Figure 2). These results highlight the need for an empirical assessment of the relative merits of the various SNP-calling methods that were published during the last two or three years (reviewed in [60]). Importantly, the two approaches used in this study yielded results reasonably consistent across species, so that the biological conclusions to be drawn (see below) are probably not method-dependent.

### Comparative population genomics in animals

The major part of the existing population genomic literature in animals is restricted to drosophila and apes. These two groups of species show contrasting patterns of within-species genetic variation, with drosophila being ∼20 times as polymorphic as humans, showing more efficient purifying selection, and higher rates adaptive evolution. Here we uncovered the population genomic profile of five new non-model species – two vertebrates and three invertebrates. These five new species appear intermediate between human and drosophila in terms of genomic diversity (Figure 4). This suggests that the typical vertebrate versus invertebrate contrast is perhaps not as sharp as suggested by the human versus drosophila comparison. So far a single species, *C. intestinalis* B, has been documented to be more polymorphic than drosophila ([17], right-most circle in Figure 4), and a single one, aye-aye, as less polymorphic than human (based on just two individuals [26]). Still, the vertebrate versus invertebrate divide is apparent in Figure 4, in which all the vertebrate species show a per-site synonymous heterozygosity below 1%, and a per-site non-synonymous heterozygosity below 6‰. This is also true of the turtle *E. orbicularis*, the single non-mammalian vertebrate included in this figure. This result appears consistent with the hypothesis that effective population size (*N*_e_) is generally higher in invertebrates than in invertebrates. The termite pattern is also quite consistent with intuitive expectations about population size: a colony of termites is comparable to many vertebrate species in terms of mass and life-history traits. Our report in termite of a significant deficit in heterogygotes (F_IS_>0.1) but no population structure (Figure S3D) is indicative of high levels of inbreeding, consistent with previous analyses in subterranean termites [61]. This tends to further reduce the effective population size in this species.

Species biology and ecology, however, does not explain every aspect of our data analysis. Hare, for instance, shows a lower π_S_ and a much higher π_N_/π_S_ ratio than rabbit, even though the two species are closely related, both phylogenetically and ecologically. The difference in π_N_/π_S_ between the two species is even stronger when our samtools-based hare estimates are considered – i.e., the very data analysis pipeline used in rabbit [24]. Similarly, *C. intestinalis* A shows evidence for a smaller population size than its sister species *C. intestinalis* B – π_S_ in A is four times as low as in B, and π_N_/π_S_ twice as high – even though the two taxa are morphologically and ecologically indistinguishable. Finally, an unexpectedly low, vertebrate-like π_S_ value is reported in flat oyster, despite the abundance of these marine animals in European Atlantic coasts

Most intriguingly, no significant difference was detected between vertebrates and invertebrates regarding the π_N_/π_S_ ratio, even though π_S_ and π_N_/π_S_ were found to be negatively correlated across vertebrates, and across invertebrates. This is paradoxical: if a population size effect indeed accounted for the negative slopes within vertebrates and within invertebrates, then why not across the whole data set? Several explanations can be suggested. First, it must be recalled that the data points in Figure 4 were taken from several distinct studies, based on distinct gene samples, and distinct data analysis methods. Perry et al. [26], for instance, only selected SNPs covered at 30X or more, equivalently to our “high-coverage” control, which yielded a slightly reduced π_N_/π_S_ ratio in ciona and hare as compared to our main analysis. It would be good to confirm the pattern of Figure 4b using a larger number species, especially non-mammals, and a common analysis strategy. Another potential methodological issue comes from our across-loci π_N_/π_S_ averaging procedure, in which mean(π_N_/π_S_) is estimated as mean(π_N_)/mean(π_S_) (see Material and Methods), which might create a downward bias of unequal magnitude among species [12].

Alternatively, the distinctive behaviour of vertebrates and invertebrates in Figure 4b might reflect a true biological difference between these two groups of species. Differences in mutation rate, hereafter noted μ, could be invoked. The π_N_/π_S_ ratio is independent of μ, whereas π_S_ is essentially proportional to μ. So if μ was generally higher in invertebrates than in vertebrates, then a higher π_S_ would be expected in the former than in the latter, for a given π_N_/π_S_ ratio. However, let us recall that what matters regarding π_S_ is the per-generation mutation rate. Published estimates of the per-generation μ indicate that this parameter is lower, not higher, in *D. melanogaster* and in the nematode *Caenorhabditis elegans* than it is in human and mouse [62], [63]. So, even though a potential influence of μ on the pattern of Figure 4b cannot be formally ruled out, current knowledge on across-species mutation rate variations would tend to even reinforce the paradox.

Selection on synonymous positions might also be a confounding factor. The genes used in this transcriptome-based study are the most highly expressed ones, i.e., prone to selection on codon usage for translation efficiency. Selected codon usage, which is documented in Drosophila but not in human [64], leads to a reduction in π_S_, and therefore an increase in π_N_/π_S_, irrespective of functional constraint on amino-acids. In mammals, synonymous positions are affected by GC-biased gene conversion [65], a neutral process that mimics natural selection, and is also expected to result in a decrease in π_S_. Substantial selective contraints on synonymous sites for efficient splicing of mRNA and nucleosome positioning are also documented, especially in mammals [66]. However, we note that such effects should affect both the X-axis (π_S_) and the Y-axis (π_N_/π_S_) of Figure 4b, so that a non-neutral behaviour of synonymous sites, if any, should essentially result in a re-scaling of the axes, not a shift upward of a subset of data points.

Another potential explanation to this unexpected pattern would invoke a difference in the selective regime between vertebrates and invertebrates. For a given *N*_e_, the π_N_/π_S_ ratio is expected to increase as the distribution of selection coefficients, *s*, of non-synonymous deleterious mutations becomes more leptokurtic [67]. One could imagine, for instance, that metabolic and protein interaction networks are more complex in vertebrates than in invertebrates [68], [69], so that the average amino-acid position is involved in a higher number of physical interactions, reducing the proportion of effectively neutral sites in vertebrates. This is consistent with the theoretical prediction of an increased variance in the distribution of deleterious selection coefficients as mutational pleiotropy increases [70]. Between-species differences in the distribution of deleterious selection coefficients are documented, with animals (drosophila and caenorhabditis) showing a higher average effect and a lower skewness as compared to micro-organisms [71].

Finally, it might be that vertebrates and invertebrates differ in their biology in such a way that the neutral and the selected levels of diversity do not respond similarly to demographic variations in the two groups. The invertebrates of this study are high-fecundity species: very large numbers of propagules (eggs, larvae, alates) are released every generation, each with a very small probability of survival to adulthood. This life cycle results in a highly skewed distribution of offspring, in which a minority of progenitors contributes to the next generation [72]. This departure from the Wright-Fisher model distinctively affects the fate of neutral [73]–[75] and selected [76] mutations, so that π_S_ and π_N_/π_S_ might respond non-linearly. At any rate, our results revivify old questions raised at the onset of experimental population genetics [77] that have been left unsolved during the long time-lag required to be able to conduct population genomics in non-model species [78].

### Concluding remarks

In this study, we showed that *de novo* population genomics in non-model taxa can be achieved based on transcriptome data. Our analysis demonstrates the contrast between vertebrates and invertebrates regarding π_N_ and π_S_, with exceptions (termites), but detects no significant difference as far as π_N_/π_S_ is concerned, questioning the hypothesis that neutral and selected levels of diversity are uniquely determined by the variations of a one-dimensional variable – i.e., *N*_e_ – across organisms. The methods developed in this study will be worth applying to additional animal species to explore further the influence of species ecology on population genomics, and the role/meaning of effective population size in molecular evolution.

## Materials and Methods

### Sampling and sequencing

Nine or ten individuals per focal species, and one to eight individuals per outgroup species, were sampled from three to ten localities across the species range. Details on sampling dates and locations are available from Table S1. Tissues were preserved from RNA degradation using liquid nitrogen, RNAlater buffer or Guanidinium thiocyanate-Phenol solution (Trizol and TriReagent BD ) was used for termites, hares and ciona. Silica membrane - SM kits (RNEasy, Qiagen) was used for hares and ciona. We previously developed a third RNA isolation method using combined GTPC and SM [79], used here for oysters and turtles. RNA quantity and quality (purity and degradation) was assessed using NanoDrop spectrophotometry, agarose gel electrophoresis and Agilent bioanalyzer 2100 system before external sequencing (GATC, Konstanz Germany). See Table S1 and reference [79] for additional details.

Five µg of total RNA of each sample were used to build 3′-primed, non-normalized cDNA libraries, sequenced using Hiseq2000 or Genome Analyzer II (Illumina) with 8 and 5 libraries pooled per lane, respectively. Fifty bp (termite) or 100 bp (other four species) single-end reads were produced. In hare, turtle and oyster, 25 µg of total RNA of one individual per focal species was used to build a random-primed normalized cDNA library. The latter was sequenced for half a run with GS FLX Titanium (Roche ). Low quality bases, adaptors and primers were removed using the SeqClean program (http://compbio.dfci.harvard.edu/tgi/).

### Bioinformatic pipeline

Figure 1 summarizes the main data analysis strategy used in this study. For each focal species, 454 and Illumina reads were assembled in contigs – i.e., predicted cDNAs – using the Abyss and Cap3 programs [80], [81], according to method D in [42]. In this approach, 454 and Illumina reads are separately assembled then merged in a mixed assembly thanks to an additional Cap3 run. Illumina reads were mapped to the contigs using BWA [82]. For each contig, average coverage was defined as the total length of mapped reads divided by contig length. Contigs less covered than an average 2.5 X per individual were immediately discarded. Open reading frames (ORF) were predicted the program transcripts_to_best_scoring_ORFs.pl, which is part of the Trinity package (http://trinityrnaseq.sf.net, courtesy of Brian Haas). This program makes use of hexanucleotide frequencies, learnt from a first pass on the data, to annotate coding sequence boundaries.

For each position of each contig and each individual, genotypes were called using the method introduced by Tsagkogeorga et al. [17] (M1 model), specifically designed to handle transcriptome-based NGS data, and implemented in the home-made program reads2snps. Briefly, this method first estimates the error rate (assumed to be shared across positions) in the maximum likelihood framework, then calculates the posterior probability of each of the 16 possible genotypes knowing the error rate, assuming Hardy-Weinberg equilibrium. When one genotype, either homozygous or heterozygous, had a posterior probability above 0.95, it was validated. Otherwise, the genotype was coded as missing data. In contrast with “variant calling” approaches (in which a homozygote is called in case of insufficient power to detect a heterozygote), no coverage-associated bias in heterozygosity prediction is expected with this method. Positions in which no more than 10 reads were available for a specific individual were also considered as missing. Prior to SNP/genotype calling, potential PCR duplicates were removed by collapsing sets of identical reads into a single read.

Paralogous gene copies are a potential source of spurious SNPs: if two distinct genes were merged in a single contig at the assembly step, then between-copy variations might be mistaken for heterozygosity. To cope with this problem, the detected SNPs were filtered for potential paralogy thanks to a newly-developed likelihood ratio test. Briefly, for a given SNP, the probability of the observed data (read counts for A, C, G and T in every individual) was calculated under the one-locus model used for SNP calling [17], on one hand, and under a two-locus model, on the other hand. The two-locus model assumes that two paralogous loci contribute reads to this SNP, with locus 1 contributing a proportion *p* of the reads. The two-locus model predicts an excess of heterozygotes (assuming that every individual carries and expresses the two loci), and correlated read count asymmetry across individuals (assuming that the relative contribution *p* of locus 1 is constant among individuals). SNPs were validated when the two-locus model did not significantly improve the fit, as compared to the one-locus model. In this test, potential departure from the 50%/50% expectation for read counts in heterozygotes was taken into account by assuming a Dirichlet-multinomial distribution of read counts, instead of a standard multinomial. Such an overdispersion of read counts is expected in case of allele-specific expression bias [83], and because of the stochasticity of allele amplification during library preparation [84]–[85]. Details of the method and simulations are provided in Text S1. The reads2snps SNP-caller and paralogue filter can be downloaded from http://kimura.univ-montp2.fr/PopPhyl/resources/tools/reads2snp.tar.gz.

Outgroup sequences were added to these alignments, when available. To achieve this aim, Illumina reads from the outgroup species were assembled using Abyss and Cap3, following method B in reference [42], and ORF were predicted as above. Orthologous pairs of coding sequences from the focal and the outgroup species were identified using reciprocal best BLAST hit, a hit being considered as valid when alignment length was above 130 bp, sequence similarity above 80%, and e-value below e^−50^. Outgroup sequences were added to within-focal species alignments using a profile-alignment version of MACSE [86], a program dedicated to the alignment of coding sequences and the detection of frameshifts. Contigs were only retained if no frameshift was identified by MACSE, and if the predicted ORF in the focal species was longer than 100 codons.

Codon sites showing a proportion of missing data above 50% were discarded. Then focal species sequences showing a proportion of missing data above 50% were removed. Alignments made of less than 10 codon sites after cleaning were removed. For each contig, the following statistics were calculated using the Bio++ library [87]: per-site synonymous (π_S_) and non-synonymous (π_N_) diversity in focal species, heterozygote deficiency (F_IS_), number of synonymous (*p*_S_) and non-synonymous (*p*_N_) segregating sites in focal species, number of synonymous (*d*_S_) and non-synonymous (*d*_N_) fixed differences between focal and outgroup species, neutrality index NI = (*p*_N_/*p*_S_)/(*d*_N_/*d*_S_) [88], and neutrality index calculated after removing SNPs for which the minor allele frequency was below 0.2 (NI_0.2_). These statistics were computed from complete, biallelic sites only – i.e., sites showing no missing data after alignment cleaning, and no more than two distinct states. The per-individual heterozygosity (proportion of heterozygote positions) was also calculated.

For each species, statistics were averaged across contigs weighting by contig length, thus giving equal weight to every SNP. Confidence intervals around estimates were obtained by bootstrapping contigs. Averaging population genomic statistics across loci can be problematic when ratios have to be calculated. The ratio of mean(π_N_) to mean(π_S_), for instance, is a biased estimate of the mean(π_N_/π_S_) if selective constraint on non-synonymous sites and neutral diversity are correlated across genes [12]. A correction for this bias was proposed [89], which is valid only if the number of synonymous SNPs per contig is large enough. This correction is not applicable to our data set, in which a majority of contigs are relatively short, and therefore include small numbers of synonymous SNPs.

The synonymous and non-synonymous site frequency spectra (SFS, i.e., the distribution of minor allele counts across SNPs) were computed based on predicted genotypes. To cope with the variable sample size across SNPs, we applied a hypergeometric projection of the observed SFS into a subsample of *n* = 12 sequences [90], SNPs sampled in less than *n* sequences being discarded. The synonymous and non-synonymous SFS were used to calculate Tajima's D [91], and to estimate the proportion of adaptive amino acid substitutions according to the method of Eyre-Walker and Keightley [53] using the DoFE program (http://www.lifesci.sussex.ac.uk/home/Adam_Eyre-Walker/Website/Software) – an estimate we call α_EWK_. This proportion was also estimated as α_0.2_ = 1−NI_0.2_ [13]. We finally calculated the (per synonymous substitution) rate of adaptive non-synonymous substitution, ω_a_ = α *d*_N_/*d*_S_ [54].

### Control analyses

Several aspects of the pipeline described above were modified in order to assess the robustness of population genetics estimates to methodological options. Here are the main alternative strategies that were explored.

#### Reference-based

In ciona and hare, illumina reads were mapped onto a reference transcriptome (downloaded from ftp://ftp.ncbi.nih.gov/genomes/ and http://www.ensembl.org/info/data/ftp, respectively, see [42]), rather than our *de novo* predicted contigs. This control is crucial in determining whether population genomics is doable in absence of a well-annotated full genome resource.

#### Threshold-free

In our main analysis, a genotype is validated when its posterior probability is above some threshold (here, 0.95). Otherwise, missing data is called. It was recently suggested that this procedure could bias allele frequency estimates [92]. In the threshold-free control, genotypes were randomly sampled according to their posterior probability, thus avoiding the use of a predefined threshold. No missing data was called provided that coverage was sufficiently high, whatever the uncertainty in genotype prediction.

#### High quality/coverage

These controls were designed to check the robustness of population genetic estimates to base call uncertainty. In the high-quality control, an initial cleaning of sequence reads was performed. For each read, the average sequence quality was computed in a 5′ to 3′, 10-bp sliding window. When a window of average quality below 30 was found, the read was trimmed by removing that window and the remaining 3′ portion of the read, thus ensuring a minimal average quality of 30 for all reads. In the high-coverage control, the required per position, per individual coverage was set to 30 X (10X in the main analysis).

#### Clip ends

Artefacts in NGS data analyses due to specific problems at the end of reads have been documented [93], [94]. Here analyses were re-conducted after removing five base pairs at both ends of all reads. This represents >10% of the total amount of data.

#### No paralog filter

In this control, the newly-introduced filter for spurious SNPs due to hidden paralogy was not applied.

#### Samtools

Our analyses were compared to an alternative SNP/genotype-calling strategy based on the algorithm implemented in samtools [49]. We followed a methodology similar to that recently published in rabbit [24]. Only SNPs with a minimum quality of 20, minimum RMS mapping quality of 20, and distancing 10 bp from indel polymorphisms were considered. Genotypes were accepted for each SNP only if sequence coverage was higher than 8X and genotype quality equal or higher than 20. Alignments were oriented and cut to the longest ORF, similarly to the main analysis. Only contigs with no frameshift and codon sites with a proportion of missing data below 50% were retained for analyses of variation.

## Supporting Information

Figure S1 Sequencing depth does not influence the estimated heterozygosity. Each dot is for an individual. Heterozygosity was calculated from both synonymous and non-synonymous positions, and averaged across contigs. Coverage was calculated after the removal of potential PCR duplicates, and averaged across contigs.
(PPT)

Figure S2 Mitochondrial DNA (*cox1*) trees for the five species analysed in this study.
Sample labels: see Table S1. Reference sequences (blue) were taken from Genbank. S2a: turtle; S2b: hare; S2c: ciona; S2d: termite; S2e: oyster.
(PPT)

Figure S3 Between-individual geographic versus genetic distances. Each dot is for a pair of individuals. X-axis: geographic distances in km; Y-axis: genetic distance, defined as (H_b_−H_w_)/H_w_, where H_b_ is the probability of drawing two distinct alleles when sampling one copy from each of the two considered individuals, and H_w_ is the average heterozygosity of the two considered individuals.
(PPT)

Figure S4 Synonymous and non-synonymous site-frequency spectra in four species.
See Figure 2 for legend.
(PPT)

Figure S5 Adaptive amino-acid substitution rate in nine animal species. From left to right: *R. grassei* (termite), *P. troglodytes* (chimpanzee), *L. granatensis* (hare), *E. orbicularis* (turtle), *O. edulis* (oyster), *O. cuniculus* (rabbit), *C. intestinalis* A (tunicate), *D. simulans* (fruit fly), *C. intestinalis* B (tunicate). π_S_ is the average synonymous diversity. *d*_N_/*d*_S_ is the non-synonymous over synonymous substitution rate ratio. α = 1−NI_0.2_ is the estimated proportion of adaptive amino-acid substitutions (low-frequency variants excluded). ω_a_ = α*d*_N_/*d*_S_ is the per synonymous substitution rate of adaptive non-synonymous substitution.
(PPT)

Table S1 Geographic origin and RNA extraction protocols for the 67 individuals analysed in this study. Preservation method: N: Liquid nitrogen; R: RNAlater buffer; G: Guanidinium thiocyanate-Phenol solution. RNA isolation method: GTPC: Guanidinium thiocyanate-Phenol Chloroform; SM: Silica membrane.
(XLS)

Table S2 Robustness of population genomic statistics to several SNP calling options.
(DOC)

Text S1 Detection of hidden paralogy in polymorphism datasets generated by mapping reads to a reference theory and simulations.
(DOC)
