# Supplementary figures and images for: Reference-Free Population Genomics from Next-Generation Transcriptome Data and the Vertebrate–Invertebrate Gap
Source: PLoS Genet. 2013 Apr 11;9(4):e1003457. doi: 10.1371/journal.pgen.1003457 (PMC3623758; doi:10.1371/journal.pgen.1003457)

## Slide 1
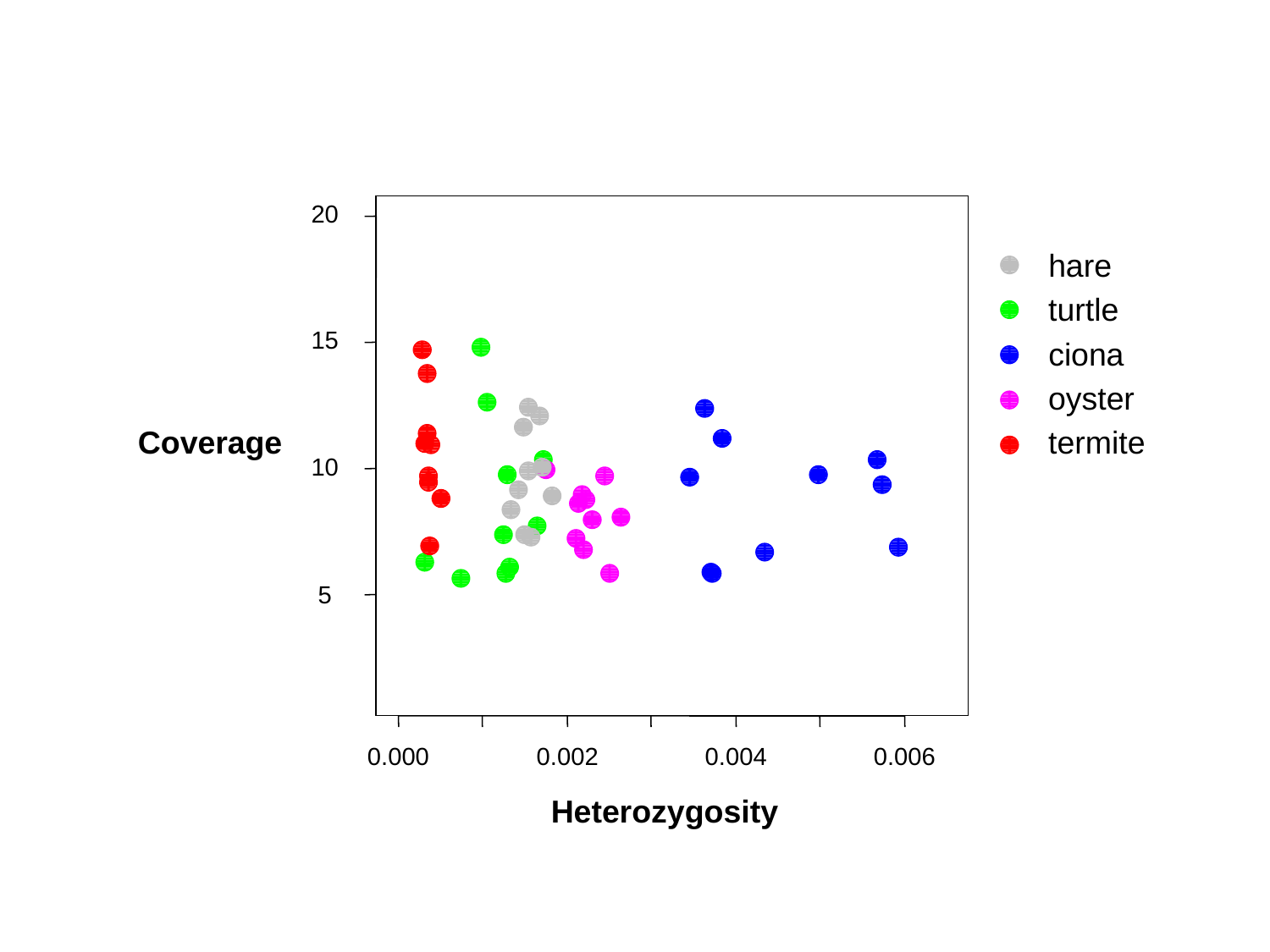

20
hare
turtle
ciona
oyster
termite
15
Coverage
10
5
0.000
0.002
0.004
0.006
Heterozygosity

Supplement: Figure S1 — Sequencing depth does not influence the estimated heterozygosity. Each dot is for an individual. Heterozygosity was calculated from both synonymous and non-synonymous positions, and averaged across contigs. Coverage was calculated after the removal of potential PCR duplicates, and averaged across contigs. (PPT) [file pgen.1003457.s001.ppt]

## Slide 1
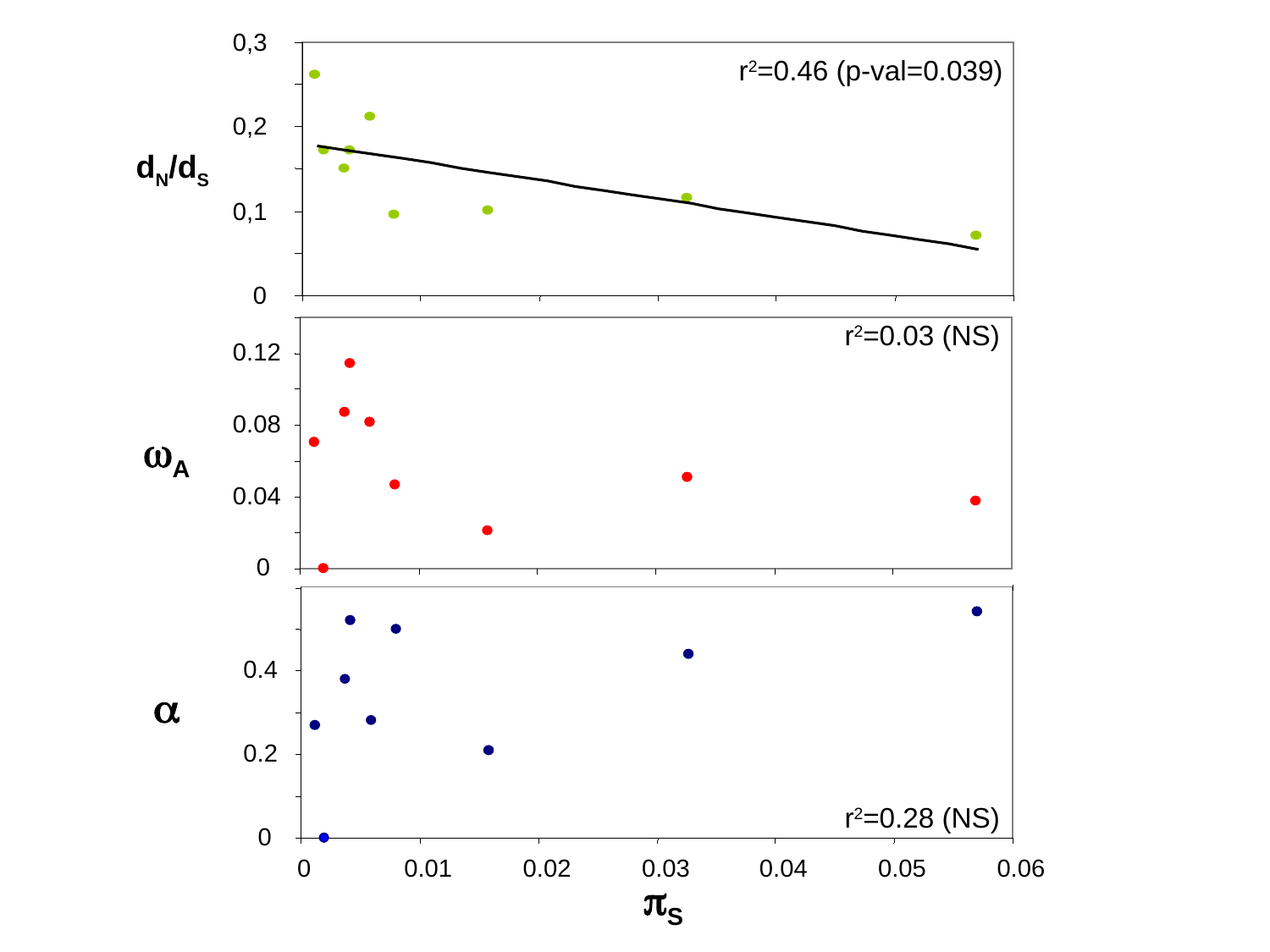

0,3
r2=0.46 (p-val=0.039)
0,2
dN/dS
0,1
0
r2=0.03 (NS)
0.12
0.08
A
0.04
0
0.4
α
0.2
r2=0.28 (NS)
0
0
0.01
0.02
0.03
0.04
0.05
0.06
S

Supplement: Figure S5 — Adaptive amino-acid substitution rate in nine animal species. From left to right: R. grassei (termite), P. troglodytes (chimpanzee), L. granatensis (hare), E. orbicularis (turtle), O. edulis (oyster), O. cuniculus (rabbit), C. intestinalis A (tunicate), D. simulans (fruit fly), C. intestinalis B (tunicate). πS is the average synonymous diversity. d N/d S is the non-synonymous over synonymous substitution rate ratio. α = 1−NI0.2 is the estimated proportion of adaptive amino-acid substitutions (low-frequency variants excluded). ωa = αd N/d S is the per synonymous substitution rate of adaptive non-synonymous substitution. (PPT) [file pgen.1003457.s005.ppt]
