# Supplementary material for: Reference-Free Population Genomics from Next-Generation Transcriptome Data and the Vertebrate–Invertebrate Gap
Source: PLoS Genet. 2013 Apr 11;9(4):e1003457. doi: 10.1371/journal.pgen.1003457 (PMC3623758; doi:10.1371/journal.pgen.1003457)
Supplement: Figure S2 — Mitochondrial DNA (cox1) trees for the five species analysed in this study. Sample labels: see Table S1. Reference sequences (blue) were taken from Genbank. S2a: turtle; S2b: hare; S2c: ciona; S2d: termite; S2e: oyster. (PPT) [file pgen.1003457.s002.ppt]

## Slide 1
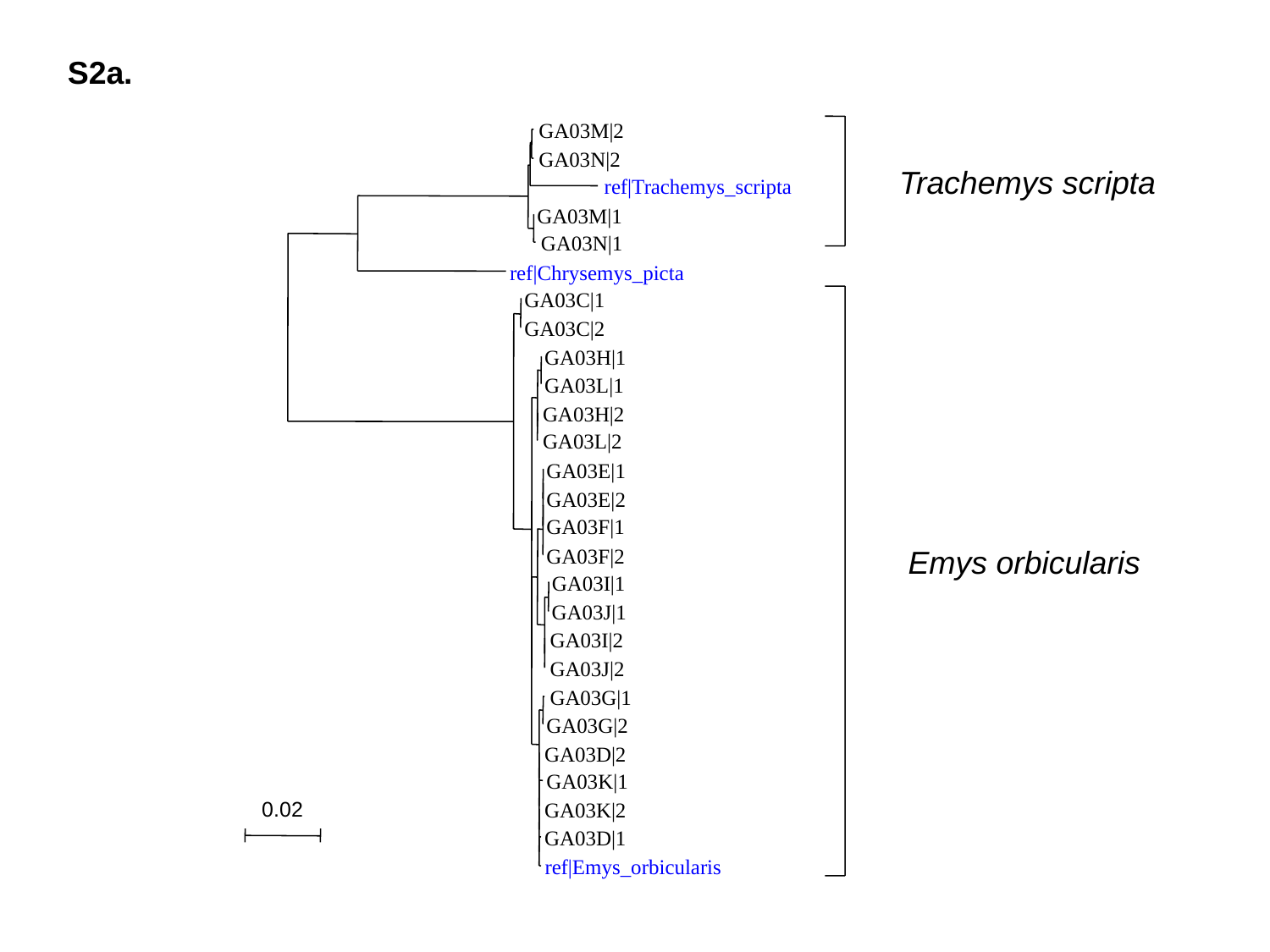

S2a.
GA03M|2
GA03N|2
Trachemys scripta
ref|Trachemys_scripta
GA03M|1
GA03N|1
ref|Chrysemys_picta
GA03C|1
GA03C|2
GA03H|1
GA03L|1
GA03H|2
GA03L|2
GA03E|1
GA03E|2
GA03F|1
Emys orbicularis
GA03F|2
GA03I|1
GA03J|1
GA03I|2
GA03J|2
GA03G|1
GA03G|2
GA03D|2
GA03K|1
0.02
GA03K|2
GA03D|1
ref|Emys_orbicularis

## Slide 2
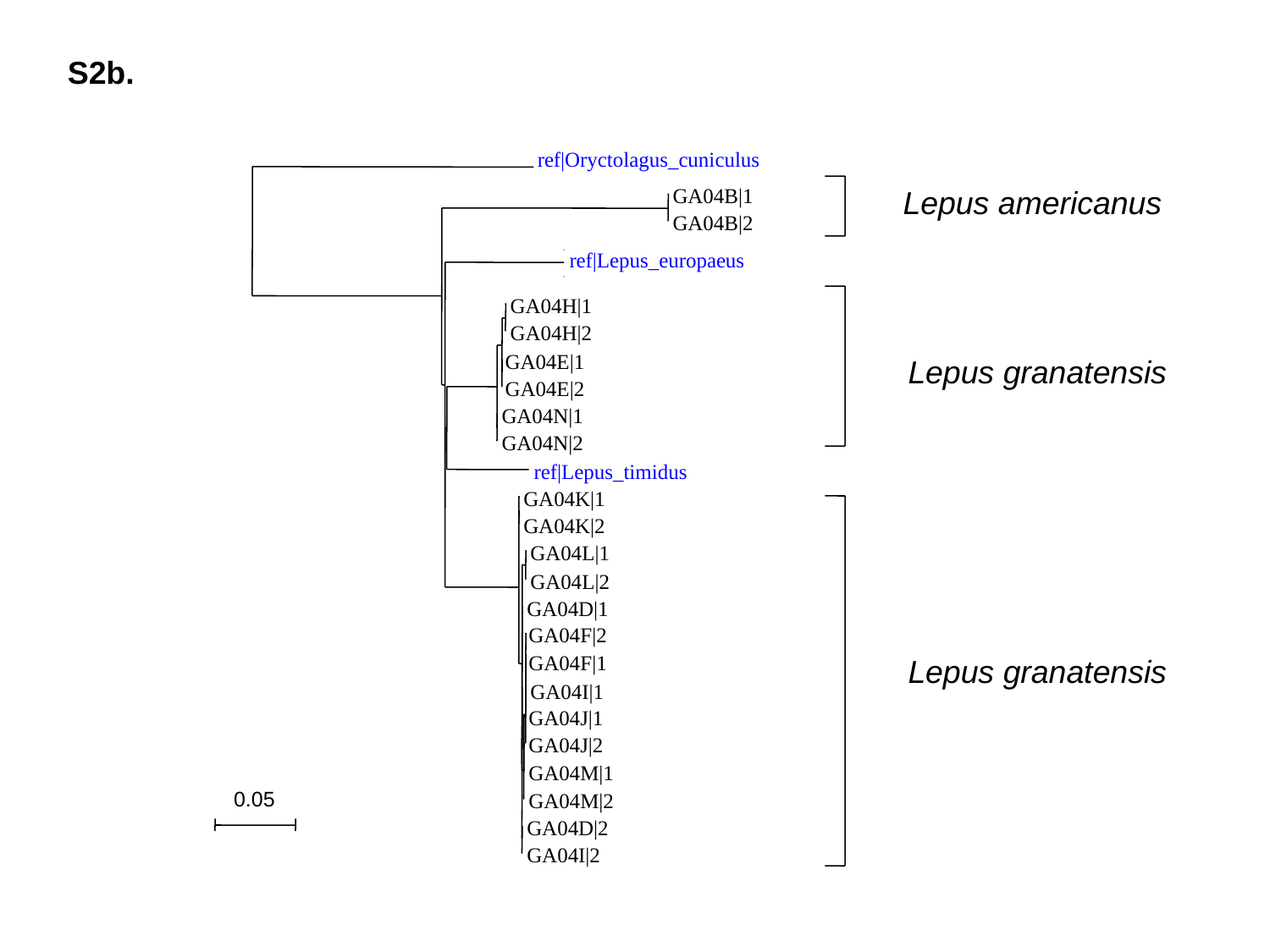

S2b.
ref|Oryctolagus_cuniculus
Lepus americanus
GA04B|1
GA04B|2
ref|Lepus_europaeus
GA04H|1
GA04H|2
Lepus granatensis
GA04E|1
GA04E|2
GA04N|1
GA04N|2
ref|Lepus_timidus
GA04K|1
GA04K|2
GA04L|1
GA04L|2
GA04D|1
GA04F|2
Lepus granatensis
GA04F|1
GA04I|1
GA04J|1
GA04J|2
GA04M|1
0.05
GA04M|2
GA04D|2
GA04I|2

## Slide 3
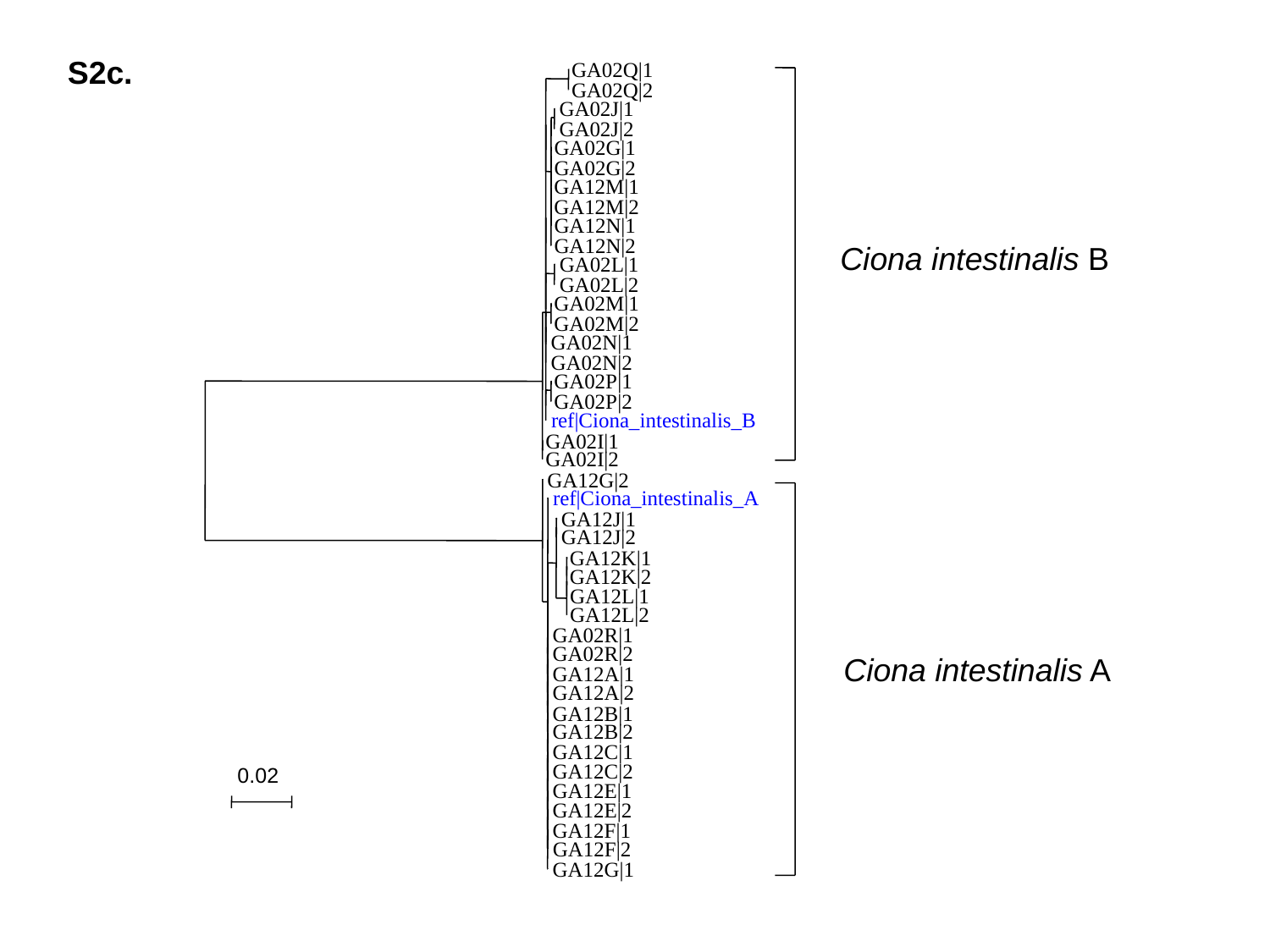

S2c.
GA02Q|1
GA02Q|2
GA02J|1
GA02J|2
GA02G|1
GA02G|2
GA12M|1
GA12M|2
GA12N|1
Ciona intestinalis B
GA12N|2
GA02L|1
GA02L|2
GA02M|1
GA02M|2
GA02N|1
GA02N|2
GA02P|1
GA02P|2
ref|Ciona_intestinalis_B
GA02I|1
GA02I|2
GA12G|2
ref|Ciona_intestinalis_A
GA12J|1
GA12J|2
GA12K|1
GA12K|2
GA12L|1
GA12L|2
GA02R|1
GA02R|2
Ciona intestinalis A
GA12A|1
GA12A|2
GA12B|1
GA12B|2
GA12C|1
0.02
GA12C|2
GA12E|1
GA12E|2
GA12F|1
GA12F|2
GA12G|1

## Slide 4
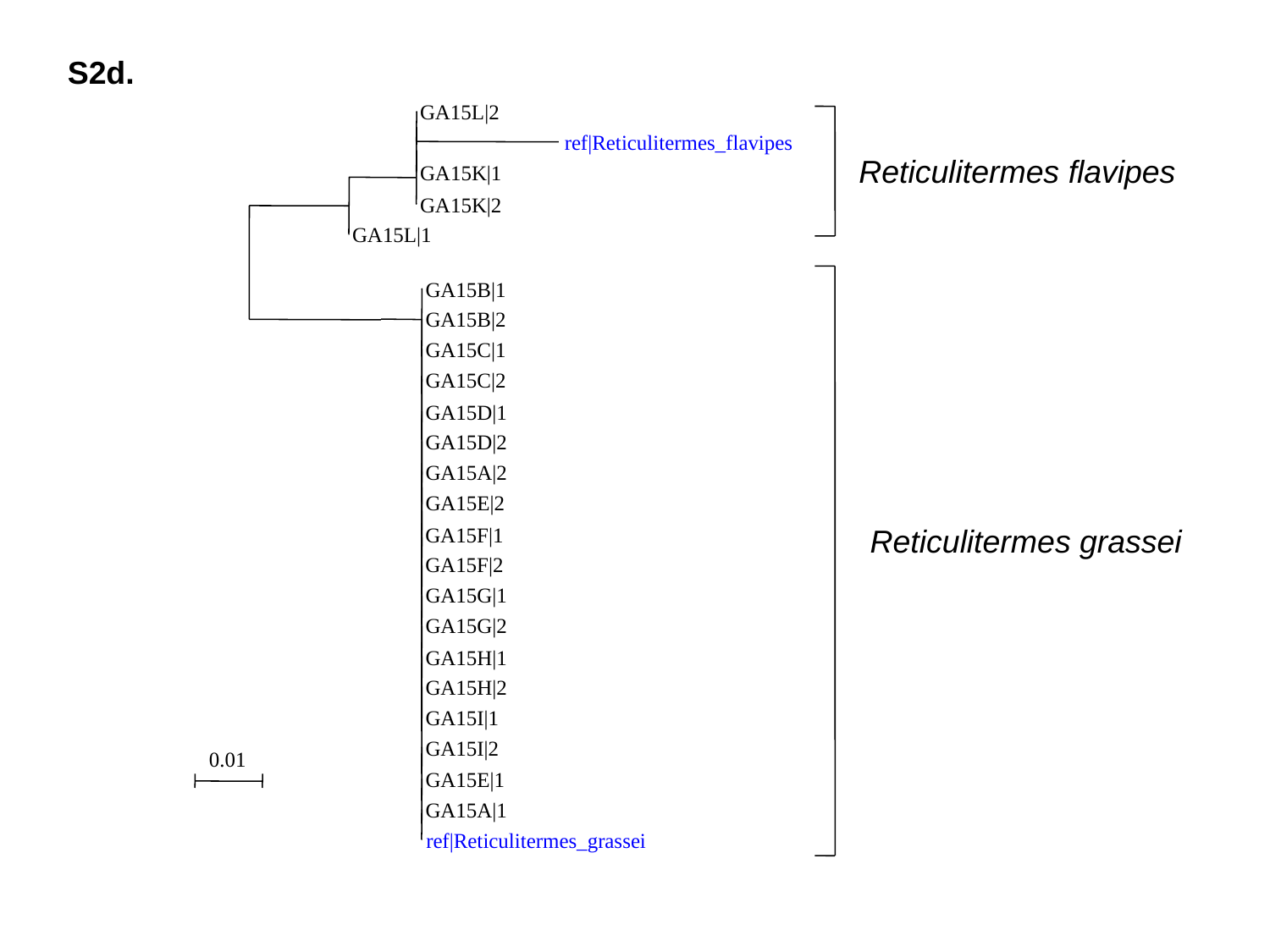

S2d.
GA15L|2
ref|Reticulitermes_flavipes
Reticulitermes flavipes
GA15K|1
GA15K|2
GA15L|1
GA15B|1
GA15B|2
GA15C|1
GA15C|2
GA15D|1
GA15D|2
GA15A|2
GA15E|2
Reticulitermes grassei
GA15F|1
GA15F|2
GA15G|1
GA15G|2
GA15H|1
GA15H|2
GA15I|1
GA15I|2
0.01
GA15E|1
GA15A|1
ref|Reticulitermes_grassei

## Slide 5
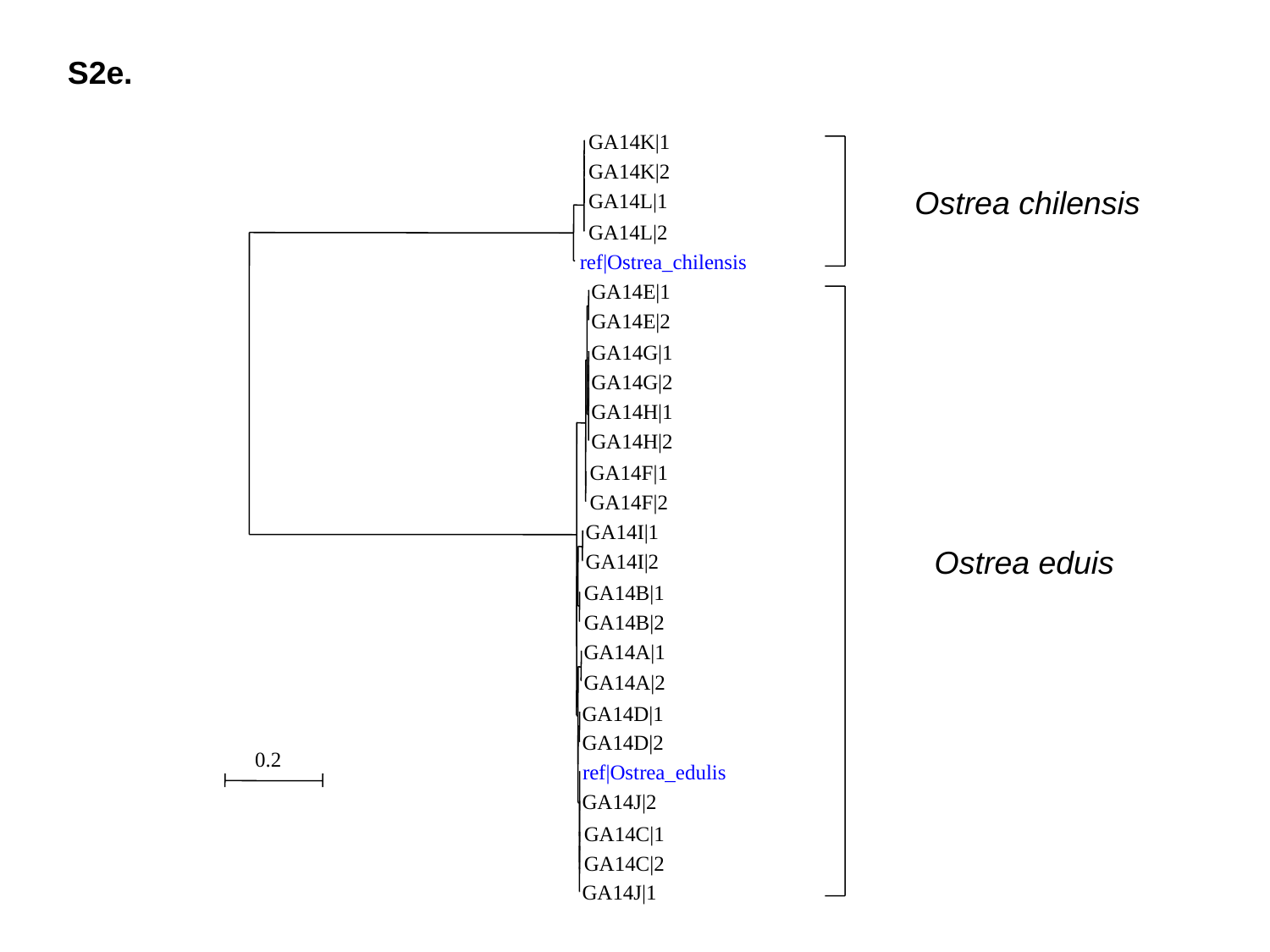

S2e.
GA14K|1
GA14K|2
Ostrea chilensis
GA14L|1
GA14L|2
ref|Ostrea_chilensis
GA14E|1
GA14E|2
GA14G|1
GA14G|2
GA14H|1
GA14H|2
GA14F|1
GA14F|2
GA14I|1
Ostrea eduis
GA14I|2
GA14B|1
GA14B|2
GA14A|1
GA14A|2
GA14D|1
GA14D|2
0.2
ref|Ostrea_edulis
GA14J|2
GA14C|1
GA14C|2
GA14J|1
