# Supplementary material for: Reference-Free Population Genomics from Next-Generation Transcriptome Data and the Vertebrate–Invertebrate Gap
Source: PLoS Genet. 2013 Apr 11;9(4):e1003457. doi: 10.1371/journal.pgen.1003457 (PMC3623758; doi:10.1371/journal.pgen.1003457)
Supplement: Figure S3 — Between-individual geographic versus genetic distances. Each dot is for a pair of individuals. X-axis: geographic distances in km; Y-axis: genetic distance, defined as (Hb−Hw)/Hw, where Hb is the probability of drawing two distinct alleles when sampling one copy from each of the two considered individuals, and Hw is the average heterozygosity of the two considered individuals. (PPT) [file pgen.1003457.s003.ppt]

## Slide 1
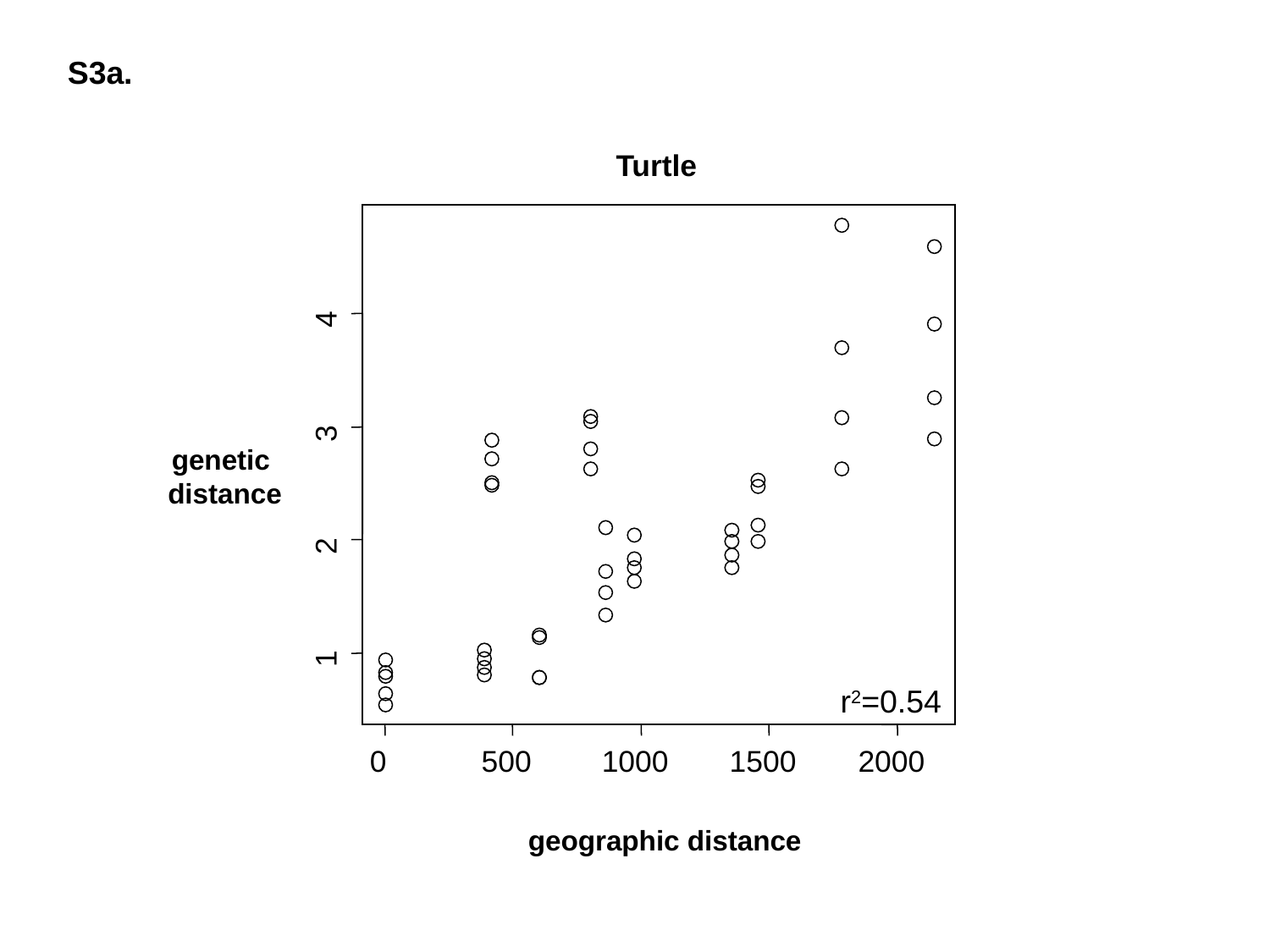

S3a.
Turtle
4
3
genetic
distance
2
1
r2=0.54
0
500
1000
1500
2000
geographic distance

## Slide 2
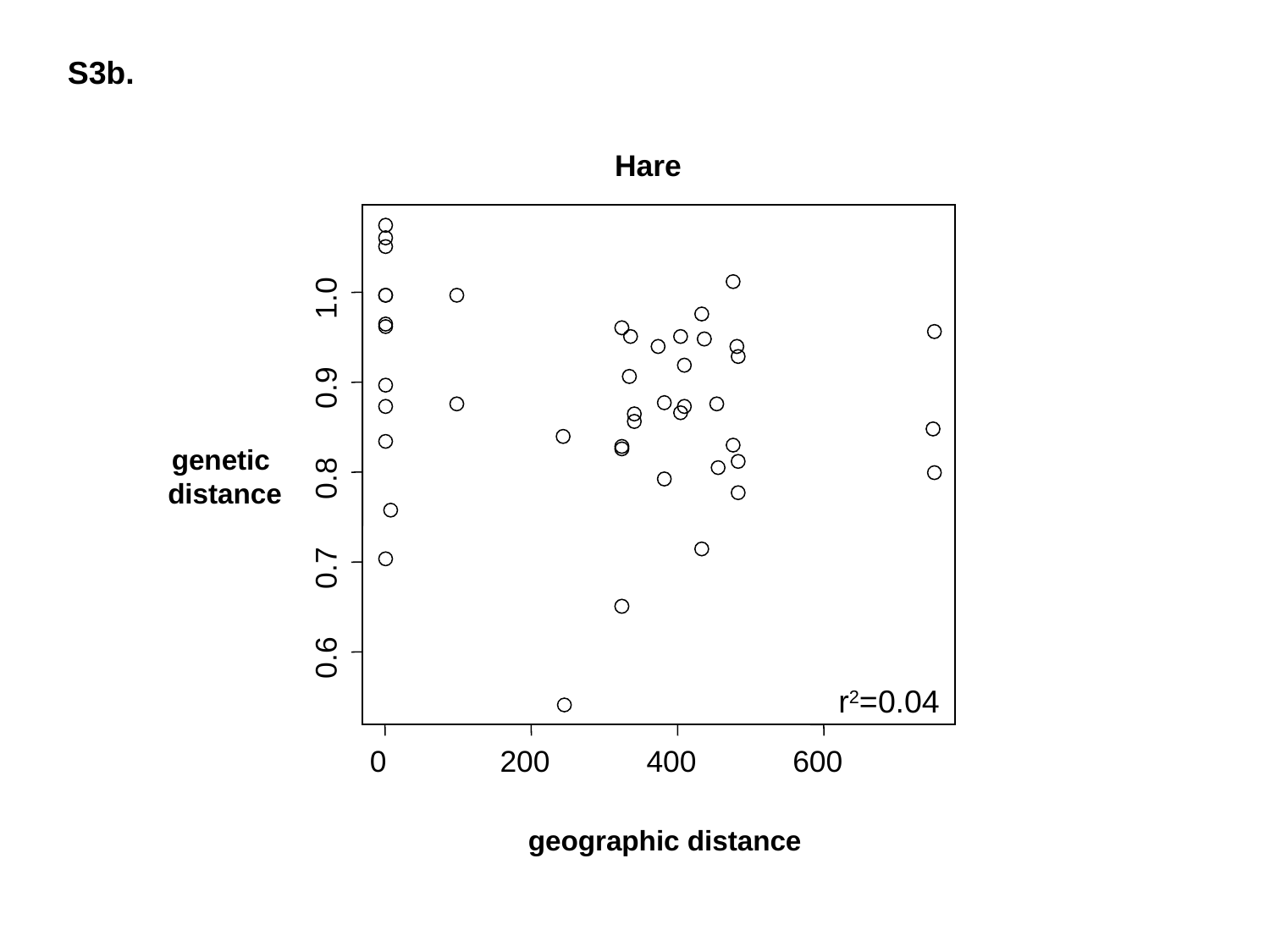

S3b.
Hare
1.0
0.9
genetic
distance
0.8
0.7
0.6
r2=0.04
0
200
400
600
geographic distance

## Slide 3
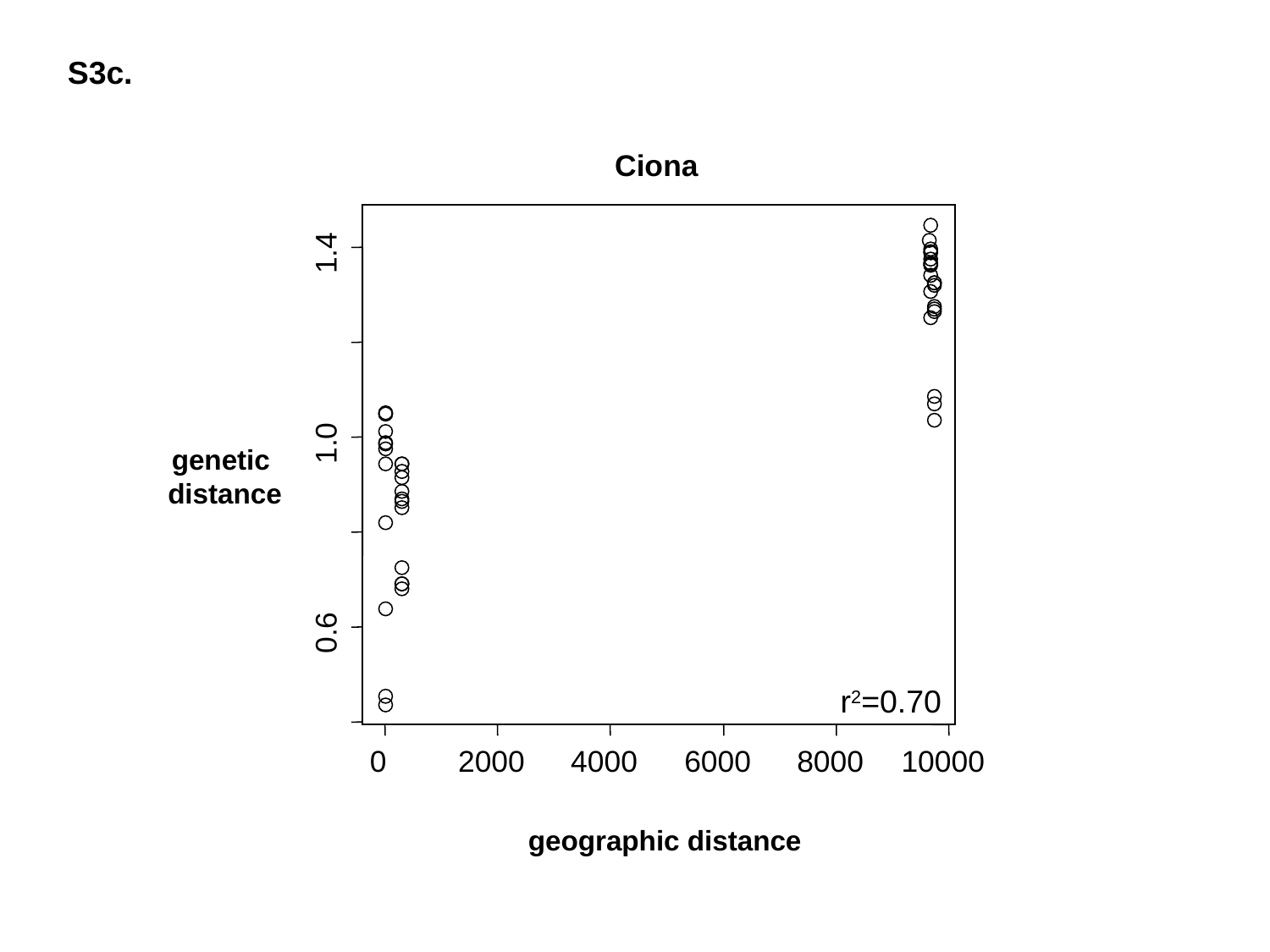

S3c.
Ciona
1.4
1.0
genetic
distance
0.6
r2=0.70
0
2000
4000
6000
8000
10000
geographic distance

## Slide 4
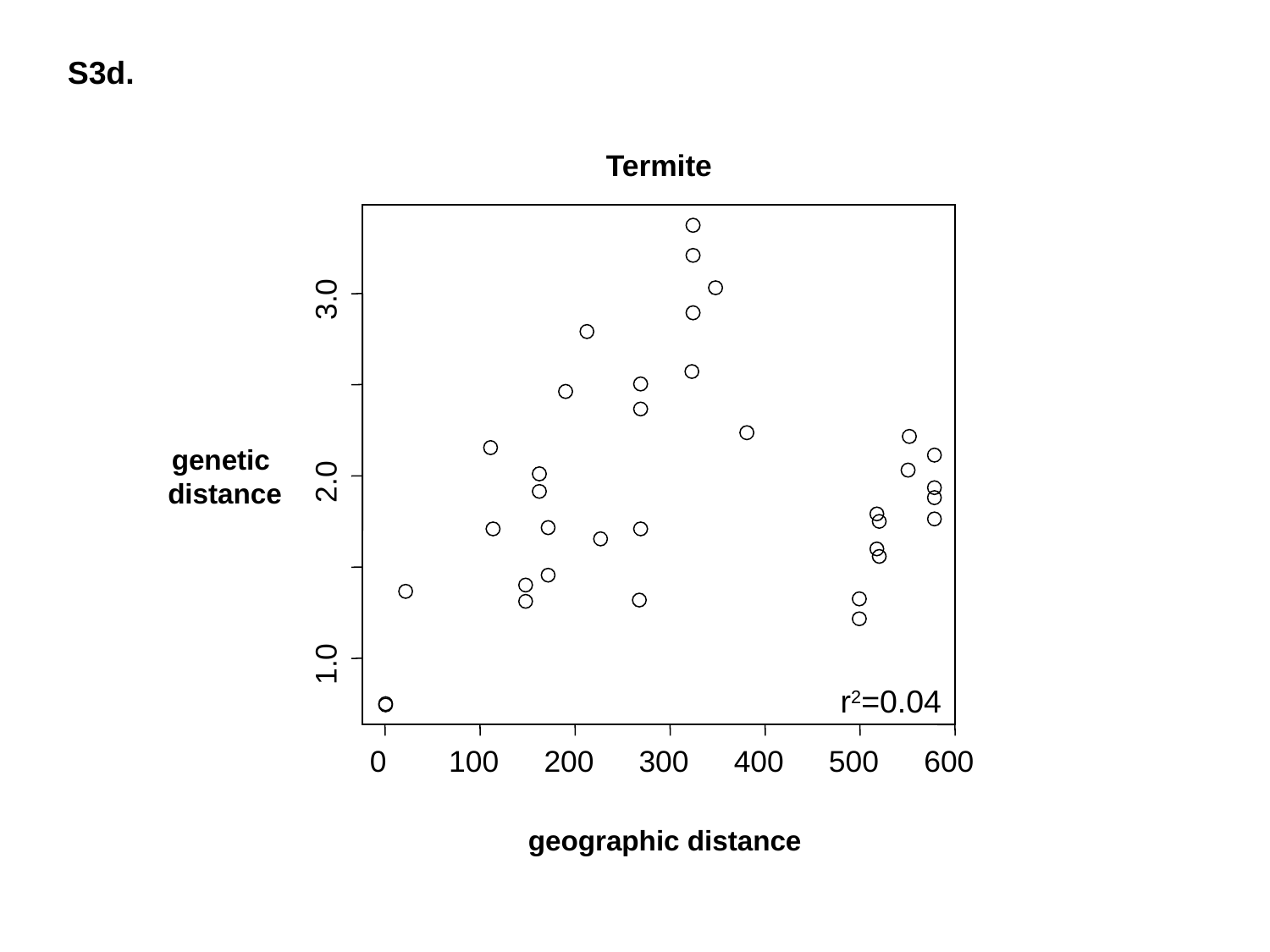

S3d.
Termite
3.0
genetic
distance
2.0
1.0
r2=0.04
0
100
200
300
400
500
600
geographic distance

## Slide 5
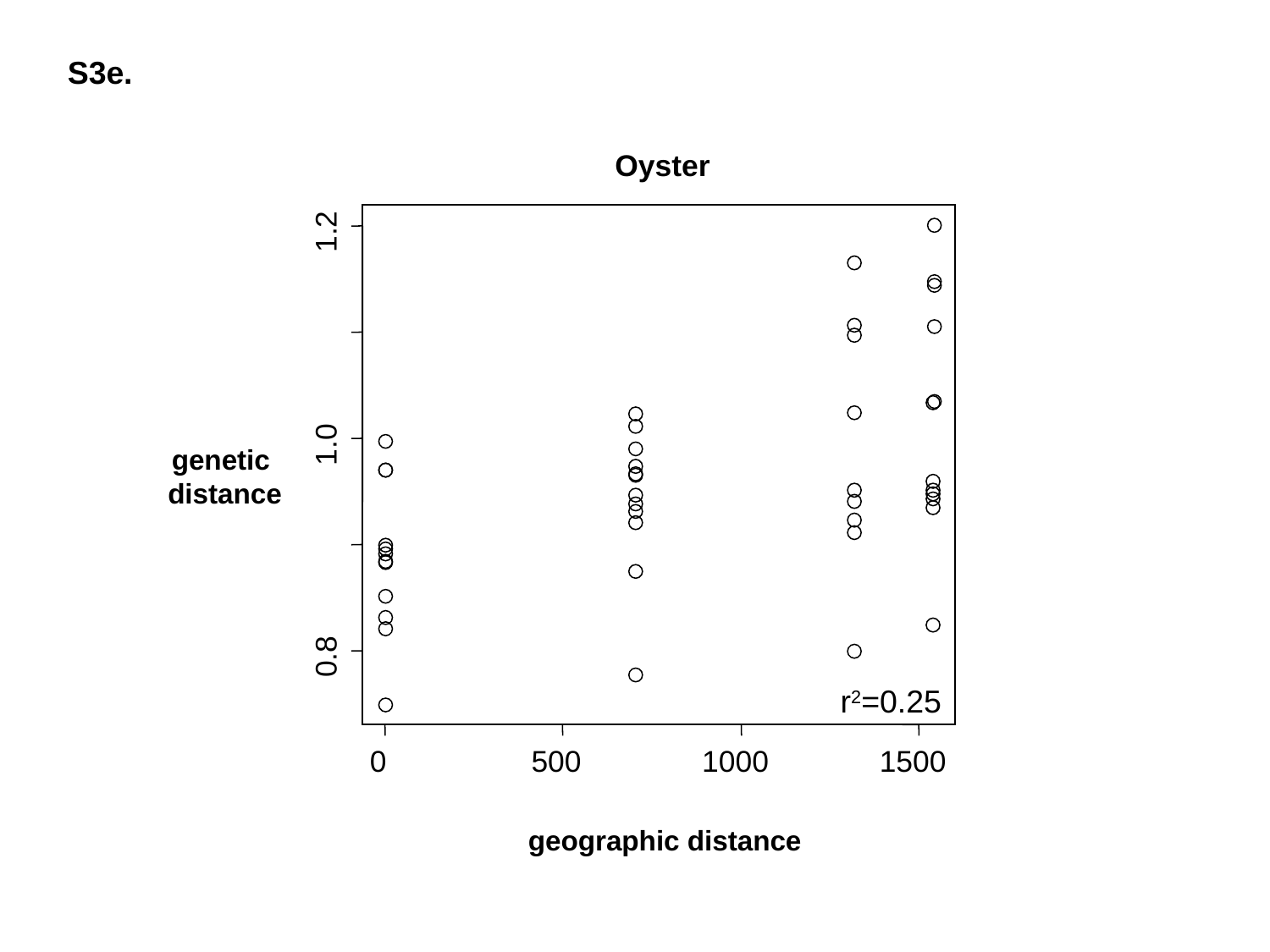

S3e.
Oyster
1.2
1.0
genetic
distance
0.8
r2=0.25
0
500
1000
1500
geographic distance
