# Supplementary material for: Reference-Free Population Genomics from Next-Generation Transcriptome Data and the Vertebrate–Invertebrate Gap
Source: PLoS Genet. 2013 Apr 11;9(4):e1003457. doi: 10.1371/journal.pgen.1003457 (PMC3623758; doi:10.1371/journal.pgen.1003457)
Supplement: Figure S4 — Synonymous and non-synonymous site-frequency spectra in four species. See Figure 2 for legend. (PPT) [file pgen.1003457.s004.ppt]

## Slide 1
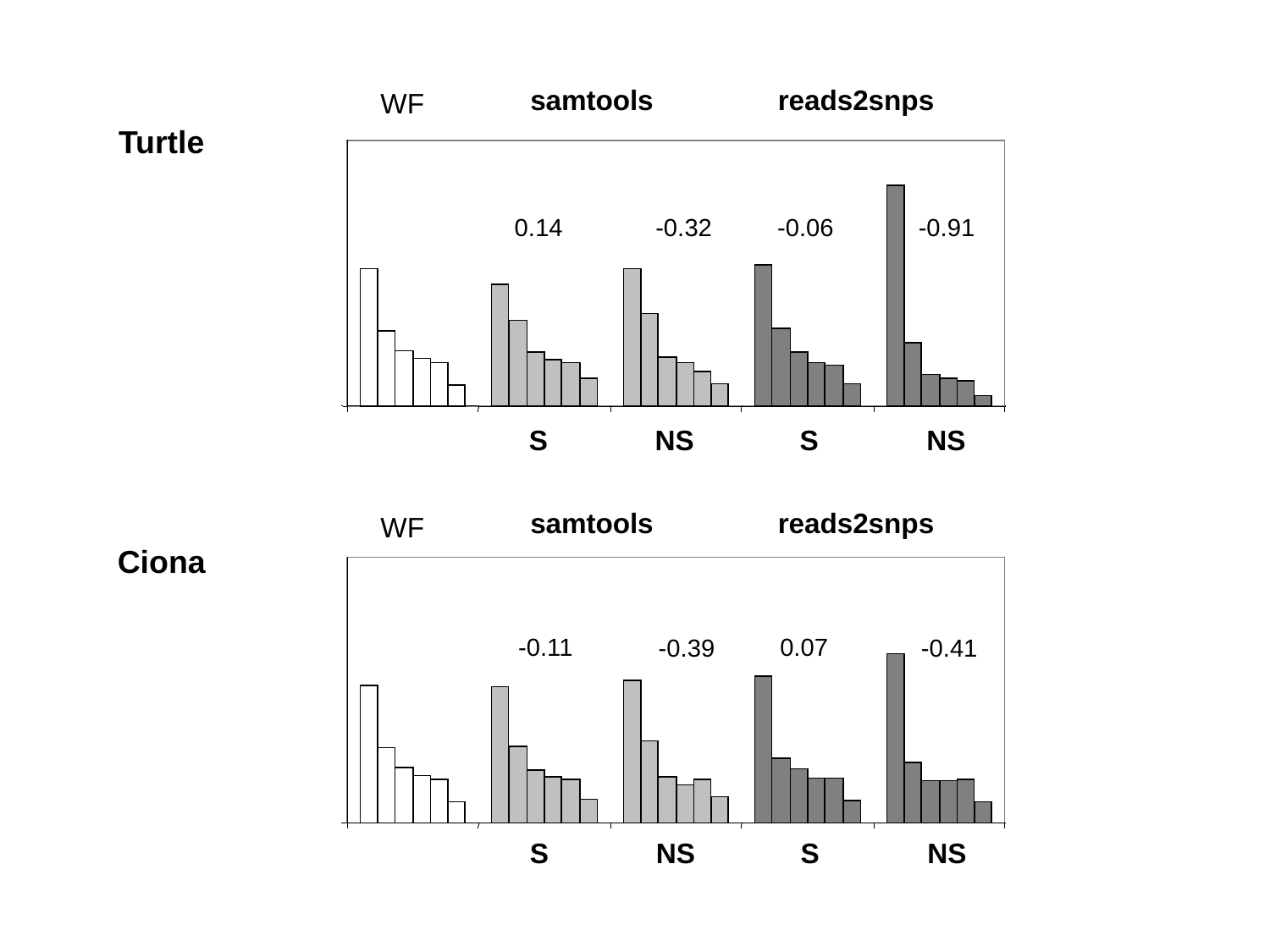

samtools
reads2snps
WF
Turtle
-0.06
0.14
-0.91
-0.32
NS
S
NS
S
samtools
reads2snps
WF
Ciona
-0.11
0.07
-0.39
-0.41
NS
S
NS
S

## Slide 2
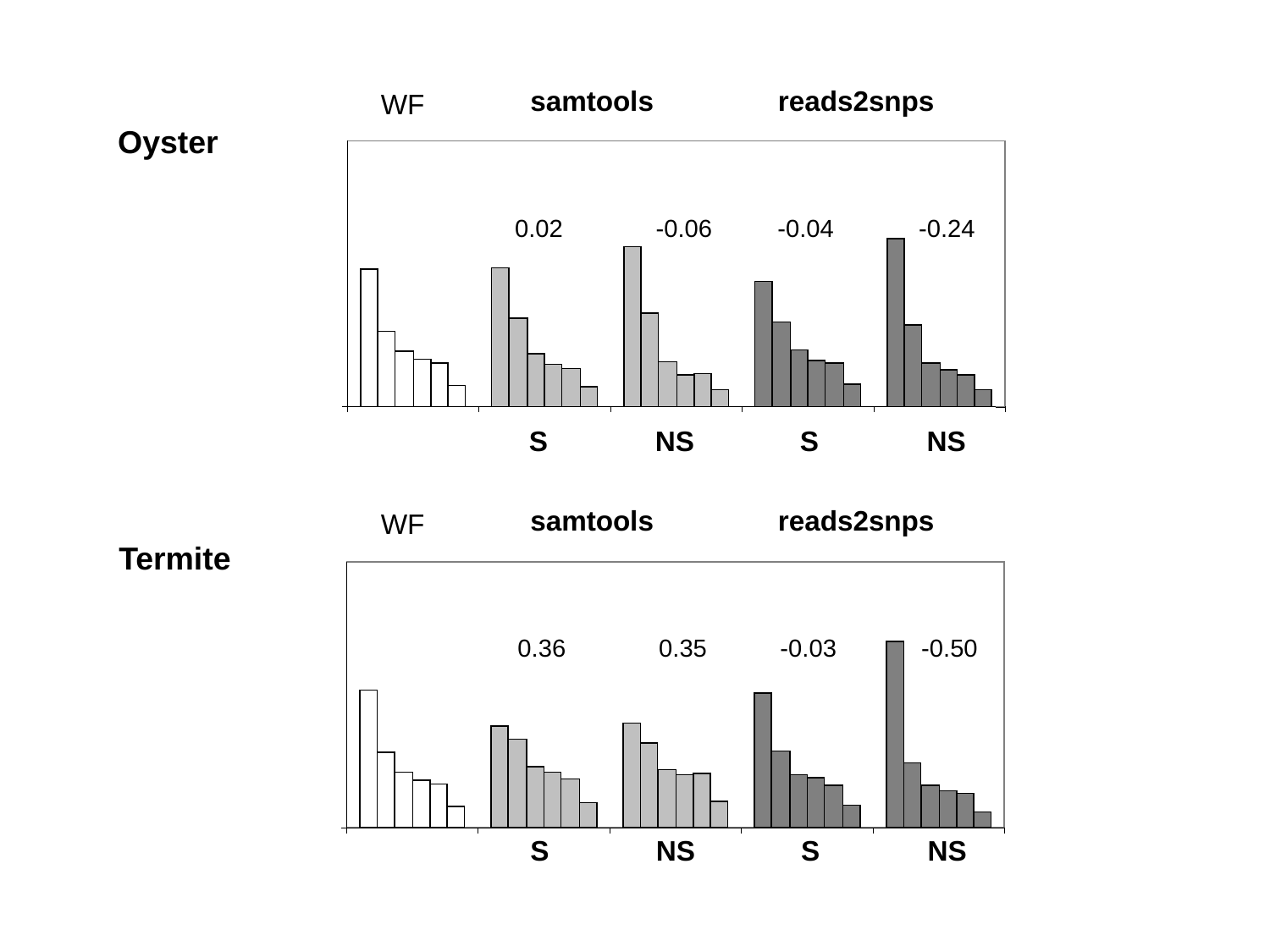

samtools
reads2snps
WF
Oyster
-0.04
0.02
-0.24
-0.06
NS
S
NS
S
samtools
reads2snps
WF
Termite
0.36
-0.03
0.35
-0.50
NS
S
NS
S
