# Supplementary material for: Reference-Free Population Genomics from Next-Generation Transcriptome Data and the Vertebrate–Invertebrate Gap
Source: PLoS Genet. 2013 Apr 11;9(4):e1003457. doi: 10.1371/journal.pgen.1003457 (PMC3623758; doi:10.1371/journal.pgen.1003457)
Supplement: Table S2 — Robustness of population genomic statistics to several SNP calling options. (DOC) [file pgen.1003457.s007.doc]

|  | #contigs | av. lg | #SNPs | π_S_ (%) | π_N_ (%) | π_N_/π_S_ | F_IS_ |
| --- | --- | --- | --- | --- | --- | --- | --- |
| **ciona**: |  |  |  |  |  |  |  |
| A. Main | 3081 | 225 | 15 826 | 1.54 | 0.17 | 0.11 | -0.04 |
| F. Threshold-free | 3 081 | 225 | 15 799 | 1.53 | 0.15 | 0.11 | -0.04 |
| G. High quality | 2737 | 222 | 13 432 | 1.58 | 0.17 | 0.11 | -0.03 |
| H. Clip ends | 2 822 | 225 | 14 096 | 1.58 | 0.17 | 0.11 | -0.03 |
| I. With outgroup | 2004 | 246 | 11 727 | 1.58 | 0.15 | 0.10 | -0.03 |
| **hare**: |  |  |  |  |  |  |  |
| A. Main | 2 624 | 276 | 7 261 | 0.41 | 0.06 | 0.15 | -0.04 |
| F. Threshold-free | 2 625 | 276 | 7 221 | 0.41 | 0.06 | 0.15 | -0.04 |
| G. High quality | 2 255 | 261 | 4 960 | 0.43 | 0.05 | 0.13 | -0.04 |
| H. Clip ends | 2 147 | 270 | 6 322 | 0.42 | 0.06 | 0.15 | -0.05 |
| I. With outgroup | 524 | 489 | 2 054 | 0.38 | 0.05 | 0.12 | -0.04 |
