# Supplementary material for: Reference-Free Population Genomics from Next-Generation Transcriptome Data and the Vertebrate–Invertebrate Gap
Source: PLoS Genet. 2013 Apr 11;9(4):e1003457. doi: 10.1371/journal.pgen.1003457 (PMC3623758; doi:10.1371/journal.pgen.1003457)
Supplement: Text S1 — Detection of hidden paralogy in polymorphism datasets generated by mapping reads to a reference theory and simulations. (DOC) [file pgen.1003457.s008.doc]

**Detection of hidden paralogy in polymorphism datasets generated by mapping reads to a reference.**

*Theory*

Genotyping methods based on read mapping typically calculate, for any given position, the likelihood of being homozygote vs. heterozygote given the observed read counts. This procedure assumes that all the reads that map to a given position genuinely correspond to this very locus. It might be, however, that reads from distinct loci map to the same place. This is expected to occur in case of undetected paralogy, copy number variation, and repetitive genomes. In this case, spurious heterozygotes will be called based on between-paralogue variation [1].

Here we introduce a test to detect these problematic cases, specifically designed for transcriptomic data. We only consider bi-allelic sites, even though the method is easily generalized to multiple alleles. The rationale is to compare the likelihood of a model assuming one bi-allelic locus with to the likelihood of a model with two bi-allelic loci, both carrying the same two alleles. In the two models, we assume Hardy-Weinberg equilibrium but it can easily be extended to more general population structure by including Wright’s fixation indices. The parameters of the two-locus model are the allele frequency at locus 1, *p*_1_ (and 1 – *p*_1_), the allele frequency at locus , *p*_2_ (and 1 – *p*_2_), the prevalence of the locus 1 relative to the locus 2, *x*, and the error rate, *ε*. For genomic data *x* should be ½. However, for transcriptomic data*, x* can take any value between 0 and 1, depending on the relative expression levels of the two paralogues. The single-locus model is simply obtained by fixing *x* = 1 Model 1 is nested in model 2, and the two models differ by two degrees of freedom.

As we only consider bi-allelic sites, we can note the vector of observed reads for a given individual, *i*, as , where *r*_1_,*_i_* is the number of reads for allele 1, *r*_2_,*_i_* is the number of reads for allele 2, and *r*_3_,*_i_* and *r*_4_,*_i_* are the numbers of reads for the two other bases, which result from sequencing errors. For a given site, the likelihoods of the two models are obtained by multiplying the likelihoods for each individual:

(1)

where *n* is the number of individuals

Model 1: a single bi-allelic locus.

Numbering the three possible genotypes – homozygote with allele 1, heterozygote, and homozygote with allele2 – as 1, 2, and 3, the likelihood for a given individual writes:

$l_{i}=\sum_{a=1}^{3} f_{a}(p)M(r_{i},q_{a})$ (2)

where *f_a_* is the probability of genotype *a*:

(3)

*M* is the probability mass function of the multinomial distribution:

$M(r,q)=\frac{R!}{\prod_{k=1}^{4} r_{k}!}\prod_{k=1}^{4} q_{k}^{r_{k}}$ with $R=\sum_{k=1}^{4} r_{k}$ (4)

with vectors of parameters:

$$q_{1}=\left\{1-3\varepsilon,\varepsilon,\varepsilon,\varepsilon\right\}$$

$q_{2}=\left\{1/2-\varepsilon,1/2-\varepsilon,\varepsilon,\varepsilon\right\}$ (5)

$$q_{3}=\left\{\varepsilon,1-3\varepsilon,\varepsilon,\varepsilon\right\}$$

Model 2: two bi-allelic paralogous loci

Now consider the model with two independent paralogues. Assuming the two loci are independent, the probability of genotype *ab* is simply given by:

(6)

and the vector of expectation for the multinomial distribution is given by:

$q_{ab}=xq_{a}+(1-x)q_{b}$ (7)

The likelihood is thus given by:

$l_{i}=\sum_{a=1}^{3} \sum_{b=1}^{3} f_{a}(p_{1})f_{b}(p_{2})M(r_{i},xq_{a}+(1-x)q_{b})$ (8)

The maximum likelihood is computed for each model and a likelihood ratio test with two degrees of freedom is performed. For this computation, *ε* is fixed to the value estimated by the reads2snps program (or any other software). Two kinds of signal help detecting paralogues, i.e., the excess of heterozygote individuals, and the systematic bias in read counts if favour of a given allele across individuals.

A potential problem with this approach is that it assumes that there is no allelic bias in heterozygotes. This could be problematic because allelic bias could increase the rate of false positive detection of paralogues. Random allelic bias seems to be frequent in NGS data and the distribution of reads often appears to be overdispersed as compared to the binomial distribution, even for genomic data[2,3]. To take overdispersion into account the previous model can be extended by using a Dirichlet-multinomial distribution instead of a multinomial distribution, which can be written as:

$DM(r,q,A)=\frac{R!}{\prod_{k=1}^{4} r_{k}!}\frac{\Gamma(A)}{\Gamma(A+R)}\prod_{k=1}^{4} \frac{\Gamma(r_{k}+Aq_{k})}{\Gamma(Aq_{k})}$ (9)

where *A* can be viewed as the overdispersion parameter, which must be estimated together with the other parameters. When *A* → ∞, the Dirichlet-multinomial distribution tends towards the multinomial distribution.

This approach is appropriate to take random allelic bias into account, i.e., when the over expressed allele is not the same one across individuals. It should be less efficient for systematic allelic bias (when the same allele is over expressed across individuals), which might be confused with the paralogue case with the Dirichlet-multinomial method.

Finally, to avoid specifying a specific alternative model, we also used a goodness of fit approach by comparing model 1 with the full model for which the expected number of reads for each base and each individual is free. This can be tested through a LRT with 3*n* – 2 degrees of freedom.

*Simulations*

We simulated datasets to assess the power and the rate of false positive of these methods. We simulated samples of 10 individuals with a coverage of 20 or 100X drawn from panmictic populations with various combinations of allelic frequencies and expression levels at the different loci. We only kept simulations with at least one individual detected as heterozygote through the *read2snp* algorithm then we applied *paraclean*.

In every simulation the individual genotypes were randomly drawn from a multinomial distribution according to their expected frequencies given by equations (3) and (6). For each genotype, the number of reads for the four bases are drawn from a multinomial distribution according to their genotype at the two loci, the proportion of the two loci, *x*, and the error rate, ε, given by equation (7).

To assess the power of the methods we first simulated the sampling of two paralogues with different allelic frequencies, *p*_1_ and *p*_2_, and relative levels of expression, *x*. Though the model assume only two paralogues, we also simulated the case of three paralogues with the two same alleles in frequency *p*_1_, *p*_2_, and *p*_3_, and proportions *x*, *y*, and (1 – *x* – *y*). We used the same procedure with equation (6) and 7 modify as follows:

(10)

and

$q_{abc}=xq_{a}+yq_{b}+(1-x-y)q_{c}$ (11)

where *a*, *b*, and *c* stand for the genotypes at the three loci. We considered cases where one locus is monomorphic and the two other are polymorphic, and the case where two loci are monomorphic for alternate alleles abd the other is monomorphic. We did not explore cases with more paralogous loci because this will increase the occurrence of three and four allelic states, which are discarded in our analysis and because with multiple loci, some will have similar allelic frequencies, which are hardly distinguishable. Remember that the aim of the test is to detect the presence of paralogues or wrong mapping, not to estimate the number of paralogues.

To asses the rate of false positive, we simulated a single locus with a fixed random allelic bias: for heterozygote individuals one of the two alleles, chosen at random for each individual, is sampled with a probability ½(1 + *b*) and the other with a probability ½(1 – *b*), where *b* is the allelic bias. In other word, reads are drawn from the multinomial distribution given by equation (5) with the *q*_2_ vector being randomly:

$q_{2}=\left\{\frac{1}{2}(1+b)(1+2\varepsilon),\frac{1}{2}(1-b)(1-2\varepsilon),\varepsilon,\varepsilon\right\}$ (12a)

or

$q_{2}=\left\{\frac{1}{2}(1+b)(1-2\varepsilon),\frac{1}{2}(1-b)(1+2\varepsilon),\varepsilon,\varepsilon\right\}$ (12b)

Note that we did not model the random allelic bias directly by drawing reads from a Dirichlet-Multinomial distribution to test whether our approach is robust to departure from the model assumptions. Nevertheless, overdispersion increases (hence *A* decreases) as *b* increases.

*Results*

As expected, under all conditions, the power was greater for the multinomial (M) test than for the Dirichlet-multinomial (DM) and goodness of fit (Gof) ones. With high coverage (100x) the rate of paralogues detection was very close to 100% under all conditions (not shown), even when the contribution of the least-expressed paralogue was very low (*x* = 0.95). With lower coverage (20x), the rate of true positive detection was high and similar for the three methods, except when the two paralogues had similar allelic frequencies, in which case the DM test had a bit less power (Figure 1). Note that the most problematic case of two homozygotes paralogues (*p*_1_ = 1 and *p*_2_ = 0) is accurately treated by the DM-test. As expected, the M and the DM tests are more efficient to detect three than two paralogues and as good or better than the GoF test (Figure 2)

Besides power, we also examined the rate of false positive detection. The powerful M test detected a high amount of false positives in case of allelic expression bias, especially with high depth of coverage (Figures 3 and 4). The Gof test also had a high false discovery rate, except for few conditions. The high rate of false positive for these two tests is due to the departure from the 1:1 allelic ratio in heterozygotes. On the contrary, the DM test showed a moderate rate of false positive detection, and thus appears as a good compromise between a sufficient accuracy to detect paralogues and a rather low rate of false positive, especially for panmictic populations. The DM test was used in the current study.

**References**

1. Dou J, Zhao X, Fu X, Jiao W, Wang N, et al. (2012) Reference-free SNP calling: Improved accuracy by preventing incorrect calls from repetitive genomic regions. Biol Dir 7:17

2. DeVeale B, van der Kooy D, Babak T (2012) Critical evaluation of imprinted gene expression by RNA-seq: a new perspective. PLoS Genet 8: e1002600.

2. Heinrich V, Stange J, Dickhaus T, Imkeller P, Krüger U et al (2012) The allele distribution in next-generation sequencing data sets is accurately described as the result of a stochastic branching process. Nucleic Acids Res 40:2426-2431.


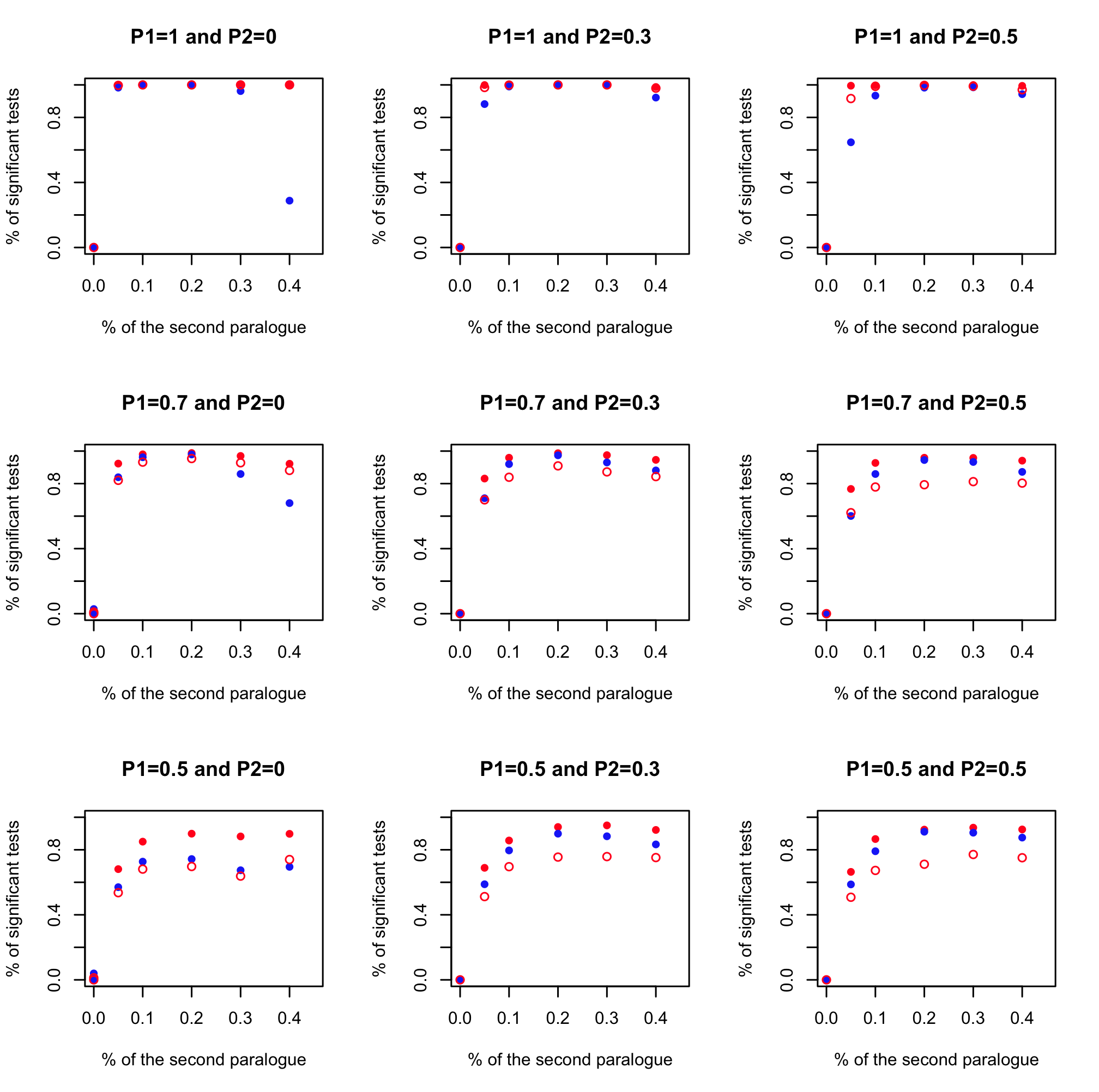


**Figure 1:** Power of the three tests to detect paralogues as function of the proportion of the second paralogues (1 – *x*) for different allelic frequencies (P1 and P2). *F* = 0. Depth of coverage = 20x. Plain red dots: M-test, Open red dots: DM-test. Blue dots: Gof-test.


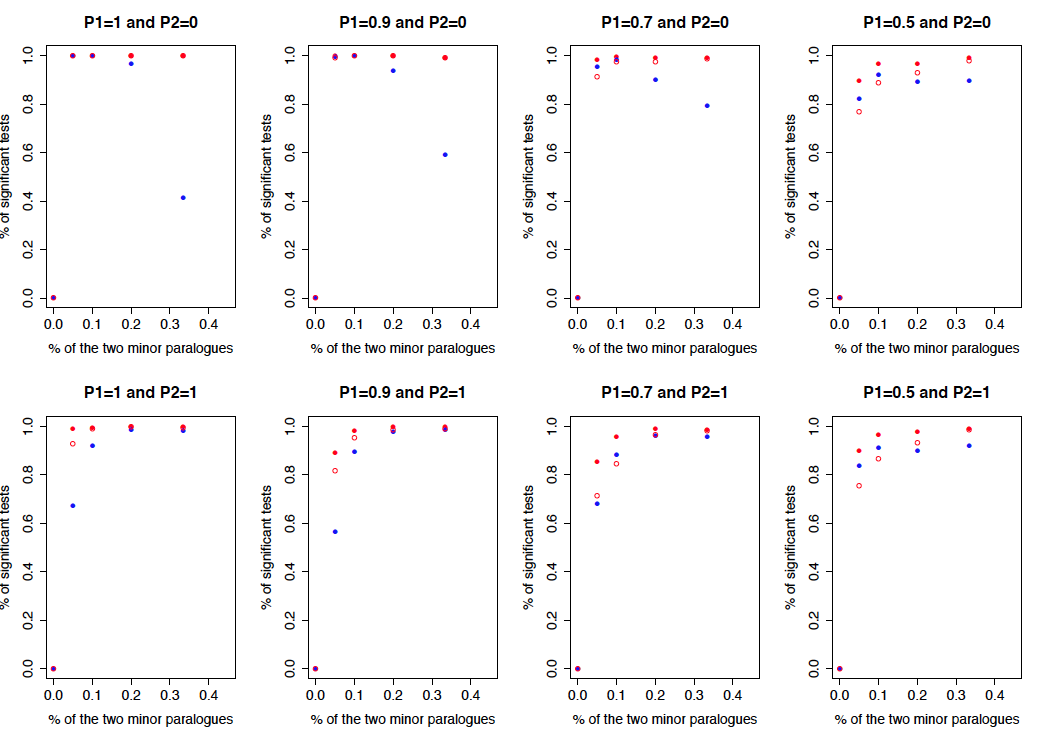


**Figure 2:** Power of the three tests to detect paralogues as function of the proportion of the minor paralogues. The two minor paralogues have the same level of expression so that the *x*-axis corresponds to (1 – *x*)/2. The proportions of the three paralogues are thus, from left to righ: 1:0:0, 0.9:0.05:0.05, 0.8:0.1:0.2, 06:0.2:0.2, and 1/3:1/3:1/3. P3 is fixed to 0.5 and P1 and P2 are indicated above each plot. Depth of coverage = 20x. Plain red dots: M-test, Open red dots: DM-test. Blue dots: Gof-test.


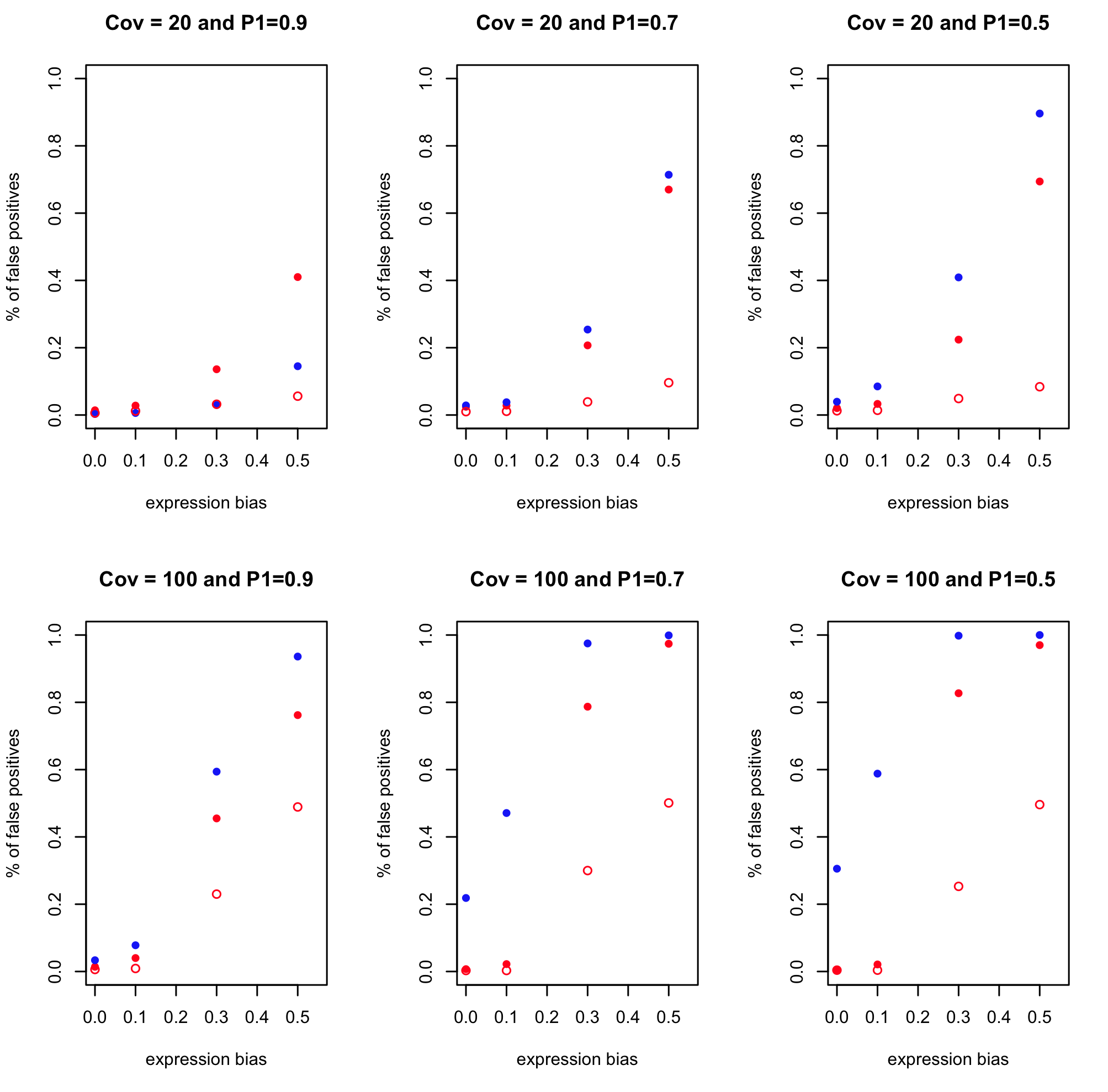


**Figure 3:** Rate of false positives as a function of allelic bias for the two depths of coverage (Cov) and different allele frequencies (P1). *F* = 0. Plain red dots: M-test, Open red dots: DM-test. Blue dots: Gof-test.
